# Spatiotemporal analysis of human intestinal development at single-cell resolution

**DOI:** 10.1016/j.cell.2020.12.016

**Published:** 2021-02-04

**Authors:** David Fawkner-Corbett, Agne Antanaviciute, Kaushal Parikh, Marta Jagielowicz, Ana Sousa Gerós, Tarun Gupta, Neil Ashley, Doran Khamis, Darren Fowler, Edward Morrissey, Chris Cunningham, Paul R.V. Johnson, Hashem Koohy, Alison Simmons

**Affiliations:** 1 Medical Research Council (MRC) Human Immunology Unit, MRC Weatherall Institute of Molecular Medicine (WIMM), John Radcliffe Hospital, University of Oxford, Oxford OX3 9DS, UK; 2 Translational Gastroenterology Unit, John Radcliffe Hospital, Oxford OX3 9DU, UK; 3 Academic Paediatric Surgery Unit (APSU), Nuffield Department of Surgical Sciences, University of Oxford, Oxford OX3 9DU, UK; 4 MRC WIMM Centre For Computational Biology, MRC Weatherall Institute of Molecular Medicine, John Radcliffe Hospital, University of Oxford, Oxford OX3 9DS, UK; 5 MRC Weatherall Institute of Molecular Medicine, John Radcliffe Hospital, University of Oxford, Oxford OX3 9DS, UK; 6 Paediatric Pathology, Department of Cellular Pathology, Oxford University Hospitals NHS Foundation Trust, Oxford OX3 9DU, UK; 7 Colorectal Surgery Department, Oxford University Hospitals NHS Foundation Trust, Oxford OX3 9DU, UK

**Keywords:** gene expression, intestinal development, human development, human developmental cell atlas, single-cell RNA-sequencing, spatial transcriptomics, mesenchymal cells, stem cells, intestinal crypt, congenital disease

## Abstract

Development of the human intestine is not well understood. Here, we link single-cell RNA sequencing and spatial transcriptomics to characterize intestinal morphogenesis through time. We identify 101 cell states including epithelial and mesenchymal progenitor populations and programs linked to key morphogenetic milestones. We describe principles of crypt-villus axis formation; neural, vascular, mesenchymal morphogenesis, and immune population of the developing gut. We identify the differentiation hierarchies of developing fibroblast and myofibroblast subtypes and describe diverse functions for these including as vascular niche cells. We pinpoint the origins of Peyer’s patches and gut-associated lymphoid tissue (GALT) and describe location-specific immune programs. We use our resource to present an unbiased analysis of morphogen gradients that direct sequential waves of cellular differentiation and define cells and locations linked to rare developmental intestinal disorders. We compile a publicly available online resource, spatio-temporal analysis resource of fetal intestinal development (STAR-FINDer), to facilitate further work.

## Highlights

• Multimodal atlas of human intestinal development maps 101 cell types onto tissue
• Charts developmental origins of diverse cellular compartments and their progenitors
• Functional diversity of fibroblasts in stem cell, vasculature, and GALT formation
• Resource applied to interrogate pathology of *in utero* intestinal diseases

## Graphical Abstract

**Figure:**
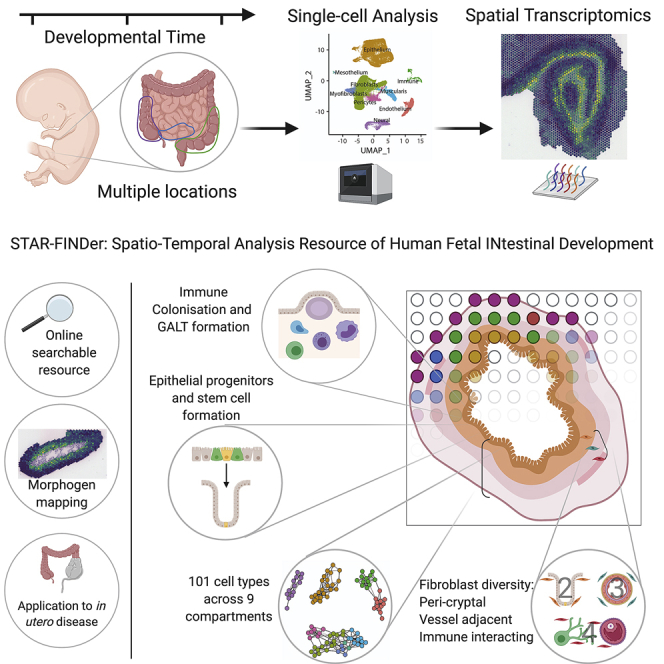

## Teaser

Fawkner-Corbett et al. chart human intestinal morphogenesis across time, location, and cellular compartments using a combination of single-cell RNA sequencing and spatial transcriptomics. The resulting online searchable atlas describes the principles of crypt-villus axis formation as well as neural, vascular, mesenchymal morphogenesis, and immune populations of the developing gut.

## Introduction

The intestine is the largest barrier organ in the body, coordinating nutritional requirements and immunity in symbiosis with intestinal microbiota (Soderholm and Pedicord, 2019). Multiple inter-related cell types constitute the mature intestine and its distinct morphology, but the molecular basis by which they develop remains unclear.

Following gastrulation, the posterior endoderm undergoes extensive folding generating the embryonic gut tube that will give rise to the small and large intestine. Within the early gut, the pseudostratified epithelial and mesenchymal cells rapidly proliferate, resulting in the elongation and widening of the gut tube. Around Carnegie stage (CS) 14, the rapidly growing intestine forms loops and subsequently (CS16) herniates into extra-embryonic coelom, to return to the abdominal cavity by 11 post-conceptual weeks (PCW). Between 8–12 PCW, the pseudo-stratified epithelium warps, generating villus and crypt structures composed of diverse differentiated epithelial cell types and establishing their self-renewing circuits maintained by epithelial stem cells (ISCs) (Gao et al., 2009; Que et al., 2007; Sherwood et al., 2009; Spence et al., 2011).

The exact molecular mechanisms driving this process in humans are not completely understood, although it is clear coordinated development between compartments is required. In mouse, PDGFRA expressing mesenchymal cells residing under primitive intestinal “hillocks” are thought to drive villus formation, whereas chick models suggest buckling forces of the developing muscularis initiate these events (Coulombre and Coulombre, 1958; Grey, 1972; Shyer et al., 2013; Walton et al., 2016). Histological analyses (Lim et al., 2020) highlight many such species-specific differences in intestinal development, underlining the need to study human tissue directly.

The intestine is a site of immune cell priming at birth (Olin et al., 2018), and defects in the ontogeny of intestinal immune cells are linked with disease (Schreurs et al., 2019). The pathogenesis of congenital or early post-natal intestinal disease is challenging to ascertain, with abnormalities occurring *in utero* when the opportunity to obtain tissue is rare.

Single-cell RNA sequencing (scRNA-seq) has facilitated the mapping of organ development at unprecedented resolution (Popescu et al., 2019) and revealed previously uncharacterized cell types and disease-associated phenotypes in the adult intestine (Kinchen et al., 2018; Martin et al., 2019; Parikh et al., 2019). Spatial transcriptomics (ST) can map transcriptional signatures to distinct geographical regions that are vital in development, where patterning and location-specific morphogen gradients shape organogenesis (Asp et al., 2019).

In this study, we exploit high-throughput scRNA-seq and ST to create a large-scale single-cell spatiotemporal atlas of human intestinal development, charting morphogenesis across time, location, and cellular compartments. We compile an integrated online resource cataloguing cellular diversity, cell-cell signaling, and transcriptional regulatory networks to highlight progenitor origins and locational fate decisions.

### Cataloguing 101 intestinal cell types across developmental time and space

**Figure 1:**
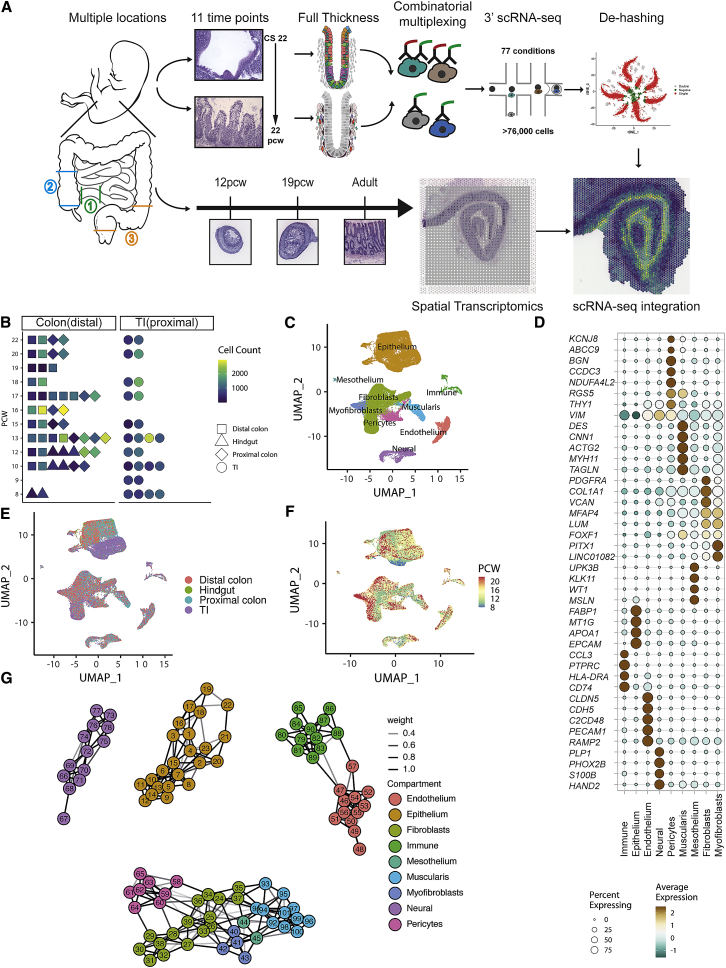
Generation of a spatio-temporal transcriptional atlas of human intestinal development (A) Overview of study design for intestinal development atlas. (B) scRNA-seq experiment sample overview dot-plot depicting sample distribution across location, developmental time and high-quality post-QC cells recovered per sample. (C) UMAP embedding of single cell transcriptomes of cells from 9 different compartments. (D) Markers of tissue compartment specific genes used for cell annotation shown as fraction of expressing cells (circle size) and mean expression (color) of gene markers (columns) across compartment (rows). (E) UMAP embedding overlay showing the location distribution across all compartments. (F) UMAP embedding overlay showing the gestational age (PCW) distribution of single cells. (G) Partition-based graph abstraction (STAR Methods) of 101 cell clusters identified in scRNA-seq data (colored by compartment, line representing weight of interaction, legend for cell cluster annotation Table S1)

**Figure S1:**
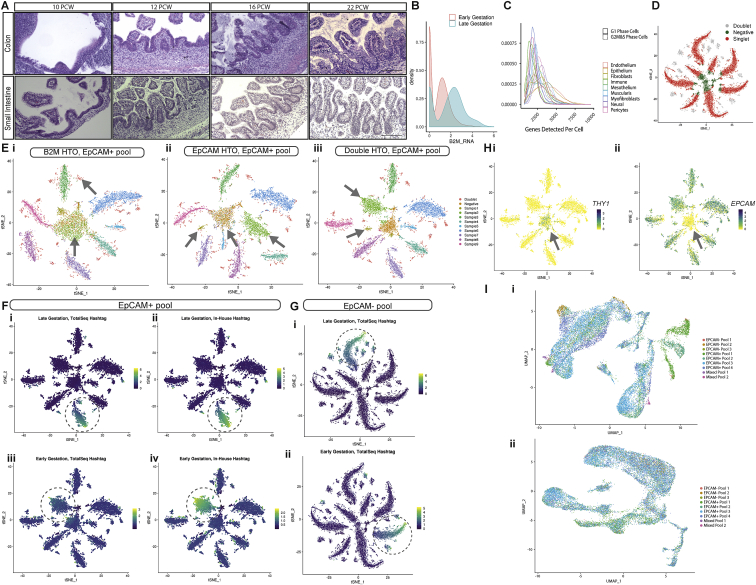
Overview of hashing methodology, sample cell-of-origin assignments and pool batch correction, related to Figure 1 and STAR methods (A) Hemotoxylin and Eosin (H&E) staining of intestinal sections demonstrating morphology of samples spanning the times and locations included in transcriptomic atlas (representative images of ≥3 samples at specified location and similar (+-1pcw) timepoints, each at 20x magnification scale bar=180 μm). (B) Example distribution of *B2M* mRNA expression in single EPCAM+ cells from an early gestation (8 PCW) sample and late gestation (19 PCW) sample from the same pool (identical sequencing depth and sample preparation conditions) showing reduced *B2M* mRNA levels in early gestation. (C) Density plot showing the distribution of per cell gene detection rate across different cell compartments. Cells are further broken down into G2M&S Phase cells (dashed line) and G1-phase cells (solid line) based on cluster analysis, as cycling cells in the G2M/S-phase tend to have substantially larger total mRNA content. (D) t-distributed stochastic neighborhood embedding (tSNE) of cells from a representative EPCAM- pool based on their recovered hashing antibody profiles, colored by classification into singlets, doublets or unstained/negative cells following dehashing (STAR methods). (E) tSNE embeddings of EPCAM+ cells from a representative pool, showing embeddings based on TotalSeq antibody tags only (i), in-house labelled antibody tags (ii) and both tags (iii). Cells are colored by sample identities assigned from dual-tag labels. Arrows indicate relevant regions highlighting multiplets (top left, (i) panel); inability to discriminate cells from low gestation samples (Sample 1 and Sample 2) when using TotalSeq tags only (center arrow, (i) panel), while the in-house tag separates cells from Sample 1 (left arrow, (ii) panel) from Sample 2 (right arrow, (ii) panel) while some untagged/negative cells still remain (center arrow, (ii) panel). (F) tSNE overlay comparing TotalSeq (i) and in-house (ii) tag signal in late gestation samples, and early gestation samples (iii-iv) in double-tagged EPCAM+ pool cells. In-house tag signal is stronger than TotalSeq tag for early gestation cells, while being comparable for late gestation cells. (G) tSNE embedding of TotalSeq tag signal in late (i) and early (ii) gestation samples in EPCAM- pools shows similar tag recoveries, in contrast to EPCAM+ pool cells in (F). (H) Expression of compartment markers for stromal cells - THY1/CD90 (i) and epithelial cells - EPCAM (ii) shown as a tSNE overlay over embedding shown in E (iii). Arrows highlight regions of interest, where most unstained/unassigned cells in EPCAM+ pools are either EPCAM- due to poor cell quality or are non-epithelial contaminants expressing stromal or immune markers (center arrow, panels i-ii). Non-epithelial contaminant cell sample-of-origin can be resolved in many cases in double-tagged pools, for instance stromal cells in central region of the embedding in other samples (Figure S1E iii). (I) Example of pool effect batch correction described in STAR methods. A UMAP embedding shows epithelial cells and their hashed pool prior to pool effect correction (i) and post-correction (ii).

We generated scRNA-seq profiles from 77 intestinal samples that were collected from 17 individual embryos representing diverse developmental time points and tissue locations (Figures 1A and 1B) (Fawkner-Corbett et al., 2020). Our dataset ranged from 8 to 22 PCW, spanning time points prior to crypt formation up to development of adult-like villus/crypt morphology (Figure S1A). We developed a full tissue sample digestion protocol with a custom multiplexing strategy using oligonucleotide-tagged antibodies (Stoeckius et al., 2018) to generate a resource of 76,592 cells (Figures 1A and S1B–S1I; STAR methods).

Clustering analyses split these into 9 intestinal compartments, annotated by transcriptional signatures—epithelial, fibroblast, endothelial (EC), pericytes, neural (ENS), muscularis, mesothelium, myofibroblast, and immune (Figures 1C and 1D)—with distinct locational and developmental time course differences (Figures 1E and 1F). Fine cluster annotation carried out based on key marker genes further identified 101 sub-populations within compartments, and we delineated their relationships using partition-based graph abstraction (Figure 1G; Table S1; STAR methods).

**Figure S2:**
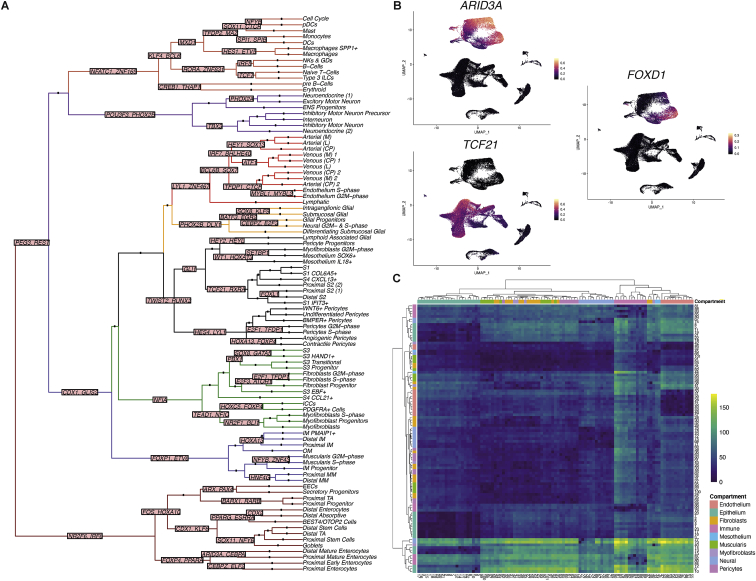
Transcription factor regulatory networks and receptor-ligand interactions in scRNA-Seq of fetal intestinal development, related to Figure 1 (A) Hierarchical clustering of all cell types based on TF module scores shows a cell type TF “decision tree”. Branches are colored by compartment. Up to top two TFs most discriminative of a branch split are shown as labels (full data for each split is provided (Fawkner-Corbett et al., 2020). (B) UMAP overlay of selected TF module AUC scores in single cells across all compartments, demonstrating gene modules with compartment specific regulation of intestinal epithelium (*ARID3A*)*,* fibroblasts (*TCF21*) and proximal and distal epithelial discriminating *FOXD1*. (C) Heatmap summarizing total cluster pairwise paracrine receptor ligand interactions. The frequency of interactions (row-wise and column wise cell type where color represents frequency; color bar indicates cluster compartment. Cluster numbers correspond to graph-abstraction in Figure 1G and Table S1)

Next, we undertook mapping of transcription factor (TF) regulatory networks to highlight key regulatory networks for each cell type and recreate a cell fate “decision tree” (Figure S2A; STAR methods). This identified 464 TF modules (e.g., *ARID3A* in epithelial development [false discovery rate [FDR] <2.2e−16, coefficient (coeff) = 0.350) and *TCF21* in fibroblasts [FDR <2.22e−16, coeff = 0.299]), recapitulated known developmental regulators (e.g., *PAX4* for enteroendocrine cell [EEC] differentiation) (Gehart et al., 2019) as well as 306 development time course and 44 location-varying regulatory networks (e.g., *FOXD1* in terminal ileum [TI] FDR <2.22e−16, coeff = 0.026, all <1% FDR, and >0.02 absolute coefficient) (Figure S2B) (Fawkner-Corbett et al., 2020). Similarly, we charted cross-talk between all 101 cell types, identifying putative receptor-ligand (RL) interactions between pairwise cell populations (up to 179 paracrine interactions per cluster pair, encompassing 2,252 RL pairs) (Figure S2C; STAR methods) (Fawkner-Corbett et al., 2020).

### Spatial location of single cells

To map spatial distributions of the scRNA-seq data, we undertook ST (Ståhl et al., 2016) on tissue from across intestinal development (8 sections from 5 samples; 12 PCW, n = 5; 19 PCW, n = 1; adult, n = 2) (Figure 1A; STAR methods).

**Figure 2:**
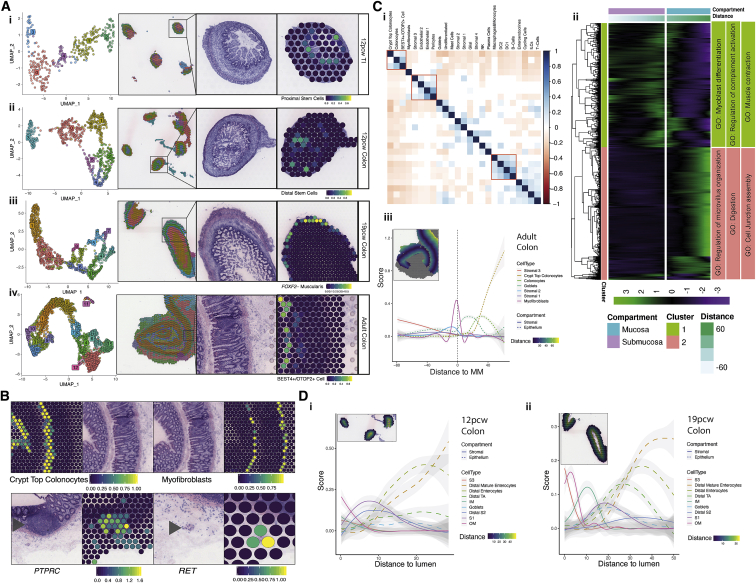
Spatio-temporal analysis of intestinal development with ST and scRNA-seq integration (A) UMAP plot of spot transcriptome clusters from each slide shown on left; clusters are visualized on tissue covered slide areas (left center). Integration with scRNA-Seq cell type annotations are shown on the right, with tissue morphology of the region shown right center for 12 PCW TI (i), 12 PCW colon (ii), 19 PCW colon (iii) and adult colon (iv) slides. All H&E images are from selected areas of ST slides from the following tissue sections: A6 (i), A8 (ii), A4 (iii) and A1 (iv). Full images are available in (Fawkner-Corbett et al., 2020). (B) Validation of ST method by comparison of adult intestinal tissue spots with histological landmarks and known related single genes – crypt top colonocyte transcriptomic signature near crypt tops (top left, left-center) and myofibroblast signature near muscularis mucosa (top right, right-center); expression of known immune cell marker *PTPRC*/CD45 in spots covering submucosal lymphoid follicle (bottom left, left-center) and expression of *RET* at myenteric plexus (bottom right, right-center). All H&E images are from selected areas of ST tissue section from section A1, H&E reference image is repeated for clarity (top). Full image is available in (Fawkner-Corbett et al., 2020). (C) (i) Pairwise cell type prediction signal correlation heatmap in adult ST spots. Non-significant correlations (<0.05 adjusted p value) are shown in white; color bar indicates Pearson’s r value. Red boxes highlight selected biologically relevant correlation groups. (ii) Heatmap showing distance-smoothed expression of significant distance-varying genes detected in adult ST slide. Vertical break indicates muscularis mucosa/distance score of zero and spots in the submucosa are assigned a negative distance score while spots in the mucosa are assigned a positive distance score. Two broad gene clusters are assigned by cutting hierarchical clustering tree, dividing the gene groups into mucosa and sub-mucosa specific expression groups. Selected GO BP terms enriched in each cluster are shown. (iii) Selected cell type prediction distribution over distance/depth score (inset and legend: ST slide overlay showing distance measures from muscularis mucosa used to assign each spot a distance gradient colored by depth score overlaid over H&E image from ST section A1, full image available in (Fawkner-Corbett et al., 2020) in adult ST showing sequential distribution of cell types, predicted using adult single cell references from (Parikh et al., 2019, GEO: GSE116222) and (Kinchen et al., 2018, GEO: GSE114374) and (Smillie et al., 2019, DUOS-000110) (D) As in (C)(iii), distance/depth score applied to fetal ST slides (inset and legend: ST slide overlay showing distance measures from serosa used to assign each spot a distance gradient colored by depth score), showing selected cell type distribution across tissue depth from serosal membrane to lumen in samples from 12 PCW colon (i) and 19 PCW colon (ii). Inset spot overlay is shown over selected areas from H&E images from ST sections A8 (i) and A4 (ii). Full images are available in (Fawkner-Corbett et al., 2020).

Analyses of transcriptional signatures of ST spots identified 5–13 spot clusters in each slide, which mapped to discrete locations (Figure 2A, STAR methods). Using our scRNA-seq atlas as a reference, we carried out factor analysis (STAR methods) to determine the likely single-cell composition of each spot, thus spatially localizing all scRNA-seq clusters. In parallel, we predicted spatial cell-type distribution in adult and fetal ST slides using adult scRNA-seq epithelial (GEO: GSE116222,GSE125970) (Parikh et al., 2019; Wang et al., 2020]), stromal (GEO: GSE114374) (Kinchen et al., 2018), and immune (DUOS-000110) (Smillie et al., 2019) data. This localized well-characterized cell types, such as ISCs at the crypt bottom (Figures 2Ai and 2Aii), *FOXF2*- muscularis cells in the outer muscle (Figure 2Aiii), and BEST4/OTOP2 cells toward the crypt top (Figure 2Aiv). Transcriptomic signatures from adult cell populations, such as crypt top colonocytes and myofibroblasts, also localized to appropriate anatomical locations (Figure 2B). Furthermore, individual gene expression aligned with morphology; *RET* showed spot-specific expression at myenteric plexuses and *PTPRC* (CD45) at submucosal lymphoid follicles (Figure 2B).

Pairwise cell-type signal correlation analysis highlighted significant same-spot co-occurrence of several cell types (e.g., BEST4/OTOP2 cells and colonocytes), in line with expected *in situ* cell localization (Figure 2Ci). Most ST spot clusters were distributed in layered ring-like formations, highlighting the largest determinant of transcriptional/cellular spatial variability corresponds to tissue depth. We identified 2,893 depth-associated genes (<5% FDR), reflecting pathways active in different layers (Figure 2Cii)—deeper spots enriched for muscular/neural processes (contraction/axonogenesis) progressing toward absorptive luminal functions (microvillus organization and digestive system processes) (STAR methods) (Fawkner-Corbett et al., 2020), in line with sequential enrichment of cell-type signatures (Figure 2Ciii). In fetal ST, notable differences included the outer and inner muscularis signatures occupying discrete spatial layers at 19, but not 12 PCW (Figure 2D).

### Human intestinal epithelial development

**Figure 3:**
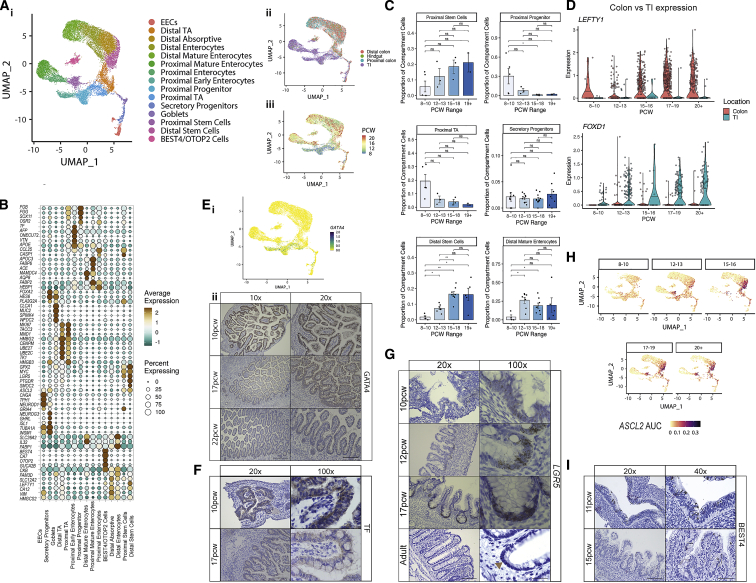
Cataloguing *in utero* epithelial maturation and crypt development (A) UMAP plot visualizing epithelial compartment populations (i) and epithelial cell distribution based on location (ii) and developmental time course (iii). (B) Dot plot of epithelial cluster markers, with color indicating average expression within cluster and dot size indicating percentage of cells within cluster expression the gene. (C) Selected population abundance changes over developmental time course shown as bar plots. Wilcox rank test, p-value < 0.05 ^∗^; p-value < 0.01 ^∗∗^; p-value < 0.001^∗∗∗^; n.s = not significant. For location-specific clusters, only location-matched samples were considered. Error bars represent standard error of the mean (SEM). (D) Violin plots showing expression of selected time-course varying genes in distal and proximal stem cells. (E) UMAP overlay visualizing expression of *GATA4* in epithelial cells (i) and representative images of SI sections from 10, 17 and 22PCW embryonic tissue stained for GATA4 by immunohistochemistry (IHC) (*n* = 3 for each individual image shown repeated on samples +-1pcw PCW, 10x/20x magnification scale bar=360/180 μm) (ii) (F) Representative images of SI sections from 10 and 17 PCW embryonic tissue stained for Transferrin (TF) by IHC (*n* = 3 on samples +-1pcw to example image, 20x/100x magnification scale bar=180/40μm) (G) Representative images of colonic sections from 10, 12 and 17 PCW embryonic tissue and adult colonic tissue stained for *LGR5* expression by single molecule in-situ hybridization (sm-ISH) (n=3 on samples +-1pcw in fetal images, 20x/100x magnification scale bar=180/40μm) (H) UMAP overlay of ASCL2 TF module AUC score over developmental time course in epithelial cells. (I) Representative images of colonic sections from 10 and 15 PCW embryonic tissue stained for BEST4 by IHC (n=3 on samples +-1pcw to example image, 20x/40x magnification scale bar=180/90μm respectively).

**Figure S3:**
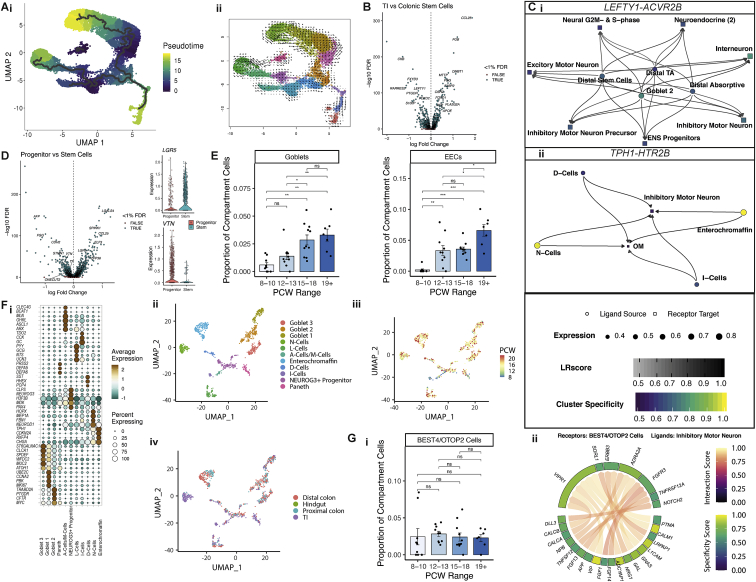
Epithelial compartment fetal intestinal development, related to Figure 3 (A) Developmental trajectory analyses of epithelial compartment cells using Monocle algorithm (i) shown over UMAP embedding, colored by pseudotime and RNA velocity estimates (ii) shown over UMAP embedding, colored by cell clusters. (B) Volcano plot (i) showing differentially expressed genes between colonic and TI stem cells. Selected genes are labelled. (C) Interacting cell network plots showing cell type cross talk via specific receptor-ligand pairs. Ligand source clusters are indicated as circles, receptor target cells as squares; autocrine interactions are shown as diamonds. Edges color indicates interaction score, node color ligand or receptor cell type specificity and node size indicates percentage of cells expressing ligand or receptor in the cluster. (D) Volcano plot (left) showing differentially expressed genes between stem cells and stem-like progenitor cells. Selected genes are highlighted. Violin plots showing stem cell-specific *LGR5* expression and progenitor specific *VTN* expression are shown on the right. (E) Goblet (left) and enteroendocrine (right) population abundance changes over developmental time course shown as bar plots. Wilcox rank test, p-value < 0.05 ^∗^; p-value < 0.01 ^∗∗^; p-value < 0.001^∗∗∗^; n.s = not significant. Error bars represent standard error of the mean (SEM). (F) Dot plot (i) heatmap showing selected epithelial secretory cell sub cluster markers. Points are scaled by percentage of cells with at least minimal (>0) detection of marker within the cluster and colored by mean cluster expression. Secretory sub-clusters are visualized as a UMAP embedding (ii), with overlays of developmental time point (iii) and location (iv). (G) BEST4/OTOP2 population abundance remains the same over developmental time course shown as bar plots (i). Wilcox rank test, p-value < 0.05 ^∗^; p-value < 0.01 ^∗∗^; p-value < 0.001^∗∗∗^; ns = not significant. (ii) Circos plot visualizing putative cross-talk between BEST4/OTOP2 cells and Inhibitory Motor Neurons. Error bars represent standard error of the mean (SEM).

*In utero* epithelial crypt formation establishes the life-long circuits for mucosal barrier maintenance (Spence et al., 2011). Our data captured 17,622 epithelial cells that were readily discriminated on their absorptive (enterocytes and BEST4/OTOP2 cells), secretory (EECs, goblet cells, and secretory progenitors), undifferentiated (distal transit amplifying [TA] and proximal TA), and stem cell (proximal and distal ISCs) gene signatures (Figure 3A; Table S1; STAR methods). Absorptive cell gene expression reflected a spectrum of maturation akin to adult epithelium (Moor et al., 2018; Parikh et al., 2019). We observed substantial locational divergence between proximal (small intestine [SI]) and distal (colonic) samples demarcated by highly specific gene expression such as *CCL25* and *APOE* (Figures 3A and 3B), confirmed by trajectory analysis and several TF modules (Figure S3A) (Fawkner-Corbett et al., 2020). This highlighted that location-specific transcriptional programs are established in development prior to crypt formation.

Substantial cellular remodeling of the epithelia was observed over time with few mature absorptive and secretory cells seen before 12 PCW, when progenitor/TA cells dominated, whereas after 12 PCW epithelial composition was already similar to adult tissue (Figure 3C).

### Developmental origins of ISCs

We observed a gradual increase in proximal and distal ISCs over time. This contrasted to early locational divergence of ISC transcriptional programs such as- e.g. distal *LEFTY1* and proximal *FOXD1* alongside their downstream regulatory network genes (Figures 3C, 3D, and S3B) (Fawkner-Corbett et al., 2020). These differences were underpinned by key locational signaling circuits, for instance, colonic *LEFTY1* participating in putative neural interactions via activin A receptor (*ACVR2B*) (Figure S3Ci).

In early development (<12 PCW), we discovered a proximal epithelial stem-like progenitor cell population, which progressed into early enterocytes. These cells exhibited many primitive features including high expression of *VTN* important in mesodermal differentiation, little *LGR5* expression compared to ISCs, and *ONECUT2* expression involved in epithelial development (Brafman et al., 2013; Dusing et al., 2010) (Figures 3B and S3D). These cells uniquely expressed TF *GATA4* in SI (Figure 3Ei), a murine early endoderm regulator (Kohlnhofer et al., 2016), the expression of which was largely lost post 12 PCW (Figure 3Eii).Transferrin (*TF*) was highly expressed by these cells (Figures 3B and 3F), confirming the importance of iron metabolism in villi formation (Anderson et al., 1991; Pu et al., 2018).

*LGR5*, a well-characterized ISC gene, was detected diffusely at low levels in early gestation (<12 PCW) across proximal/distal ISCs and stem-like progenitors (Figures 3B and S3D). *In situ* hybridization (ISH) confirmed diffuse *LGR5* expression at 10 PCW that later localized to the crypt base (Figure 3G), analogous to behavior of *Lgr5* reported in chick and mouse development (Shyer et al., 2015). Even after crypt morphology was established (e.g., post-19 PCW), ISCs constituted a mean of 18%–22% of captured epithelial cells in distal/proximal samples, respectively (Figure 3C), higher than 3%–4% captured in scRNA-seq studies of adult colon (Parikh et al., 2019; Smillie et al., 2019). In line with this, we found a significant increase in *ASCL2* TF module (encompassing downstream target *LGR5*) over developmental time (Figure 3H) (Fawkner-Corbett et al., 2020).

### Specialized neural-epithelial circuits preface ISC development

Secretory lineage cells arose by 12 PCW, with few goblets detected at 8–10 PCW; however, there was a gradual increase in goblets and EECs post-12 PCW reflecting ongoing maturation (Figures 3A and S3E). Secretory cells further subdivided into 11 clusters reflecting distinct EEC subtypes (A/M, D, and Enterochromaffin/I/L/N-cells) alongside SI-specific Paneth cells, goblet cells, and a *NEUROG3*^*+*^ progenitor population (Figure S3F; Table S1; STAR methods) (Beumer et al., 2020). Unlike other EEC subtypes I- and A-cells were detected earlier, alongside Paneth cells (Figure S3F).

RL mapping established core EEC signaling circuits such as Enterochromaffin cells, mainly seen in colon from 12 PCW, interacting with inhibitory motor neurons through numerous pathways including *TPH1-HTR2B*, a known serotonin signaling pathway in appetite/motility (Figure S3Cii). Thus, EEC diversity and related networks are largely established alongside crypt formation.

In contrast, BEST4/OTOP2 cells appeared at the earliest time points prior to crypt formation, were already transcriptionally distinct (Figure 3I), and showed no changes in frequency over time (Figure S3Gi), suggesting their development may be uncoupled from normal crypt-villus circuitry. Furthermore, RL analysis predicted a strong interaction between BEST4/OTOP2 and neuronal cells (also established early in development); e.g., inhibitory motor neurons expressing neurotransmitter *VIP* that mediates secretion through receptor *VIPR1* expressed on BEST4/OTOP2 cells (Figure S3Gii).

### Cross-compartmental coordinated development

Intestinal development requires precise interaction between cell types from all three germ layers (de Santa Barbara et al., 2003). We identified 78 non-epithelial cell clusters across 8 compartments, classified based on their transcriptional, temporal, and locational profiles (16 fibroblast, 4 myofibroblast, 2 mesothelial, 12 EC, 8 pericyte, 13 neural, 12 immune, and 11 muscle) (Table S1; STAR methods). Across these cells, we observed coordinated dynamics of differentiation, with some populations appearing first and thus likely establishing critical niches that pave way for other cells, whereas, in others, co-localizing populations evolved in tandem.

### Establishment of intestinal angiogenesis

**Figure 4:**
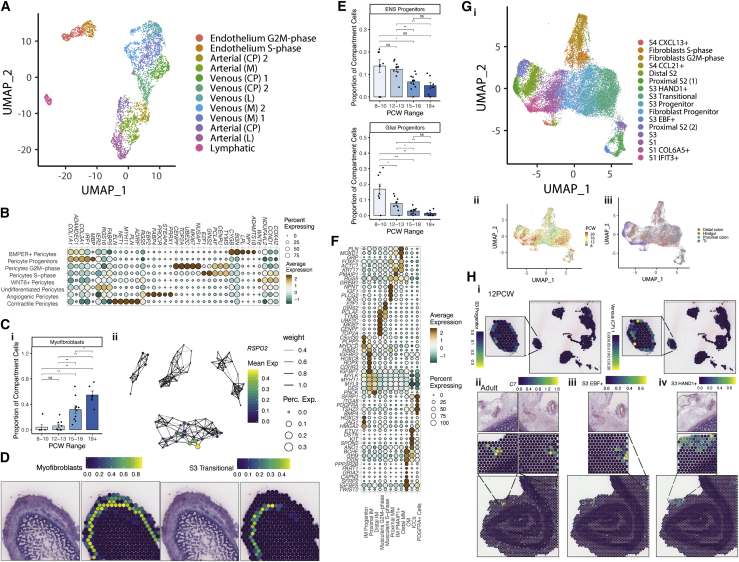
Coordinated development of mesenchymal and endothelial compartment cell (A) UMAP visualization of endothelial compartment subclusters. (B) Dot plot heatmap showing selected pericyte cell sub-cluster markers. Points are scaled by percentage of cells with at least minimal (>0) detection of marker within the cluster and colored by mean cluster expression. (C) (i) Mature myofibroblast population abundance changes over developmental time course shown as bar plots. Wilcox rank test, p-value < 0.05 ^∗^; p-value < 0.01 ^∗∗^; p-value < 0.001^∗∗∗^; ns = not significant. Error bars represent standard error of the mean (SEM). (ii) Partition-based graph abstraction showing expression of myofibroblast marker *RSPO2*. (D) ST spot overlay of cell type predictions for myofibroblast cells and S3 transitional at 19 PCW. All H&E images and corresponding spot overlays show selected area (image repeated for clarity) from ST tissue H&E image section A4. Full image is available in (Fawkner-Corbett et al., 2020). (E) ENS and glial progenitor population abundance changes over developmental time course shown as bar plots. Wilcox rank test, p-value < 0.05 ^∗^; p-value < 0.01 ^∗∗^; p-value < 0.001^∗∗∗^; ns = not significant. Error bars represent standard error of the mean (SEM). (F) Dot plot heatmap showing selected muscle cell sub-cluster markers. Points are scaled by percentage of cells with at least minimal (>0) detection of marker within the cluster and colored by mean cluster expression. (G) (i) UMAP overlay visualizing fibroblast sub-populations. UMAP overlay showing the distribution of fibroblast cells over developmental time (ii) and location (iii). (H) Co-localization of S3 progenitor cells and venous (CP) cells in 12 PCW ST slide (i); Co-localization of S3 marker C7 with large vessels in adult ST slide (ii) and localization of S3 EBF+ (iii) and S3 HAND1+ (iv) cells to areas surrounding vessels in adult slides. H&E image spot overlays show selected areas from ST tissue H&E image sections A8 (i) and A1 (ii-iv). H&E reference image repeated for clarity at each zoomed in location. Full images are available in (Fawkner-Corbett et al., 2020).

**Figure S4:**
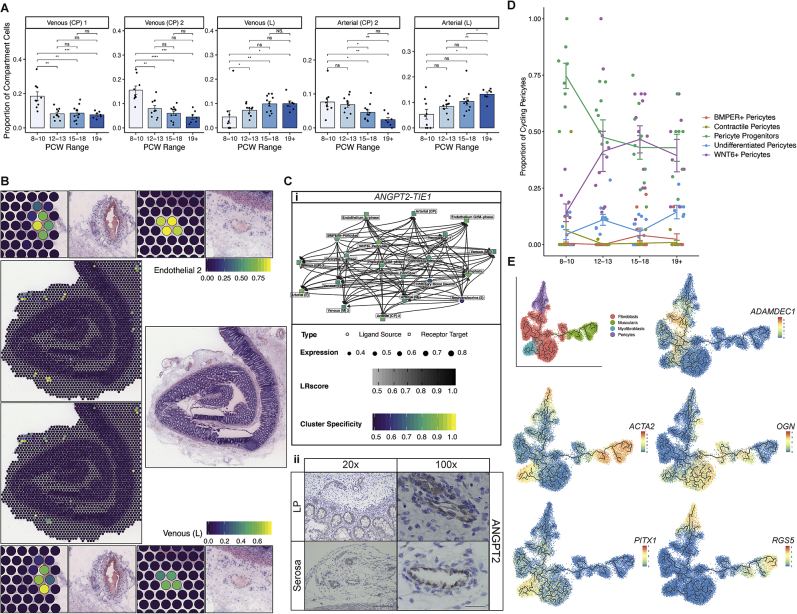
Development of endothelial and pericyte compartment cells, related to Figure 4 (A) Selected endothelial population abundance changes over developmental time course shown as bar plots. Wilcox rank test, p-value < 0.05 ^∗^; p-value < 0.01 ^∗∗^; p-value < 0.001^∗∗∗^; ns = not significant. Error bars represent standard error of the mean (SEM). (B) ST spot cell type predictions of endothelial cluster 2 from single cell reference in (Kinchen et al, 2018) (top) and large venous single cell signature from scRNA-seq data here shown in adult slides (bottom). Areas with vessels are zoomed in for clarity. All H&E images and corresponding ST spot overlays are plotted over ST H&E section A1, reference H&E images are repeated for clarity (top and bottom zoom). Full image is available in (Fawkner-Corbett et al., 2020). (C) Interacting cell network plot (i) showing cell type cross talk via specific receptor-ligand pair, ANGPT2 and TIE1. Ligand source clusters are indicated as circles, receptor target cells as squares; autocrine interactions are shown as diamonds. Edges color indicates interaction score, node color ligand or receptor cell type specificity and node size indicates percentage of cells expressing ligand or receptor in the cluster. (ii) Representative images of late (19–22 pcw) colonic sections stained for ANGPT2 protein by IHC with positive expression around vessels observed in the lamina propria (LP) and serosa (n=4 experiments on individual samples 19–22pcw, 20x/100x magnification, scale bar=180/40μm). (D) Time course abundance changes in S- and G2M- phase pericytes, highlighting proliferating dynamics of pericyte compartments. Abundance changes in cycling and non-cycling counterparts of pericyte progenitors, WNT6+ pericytes and contractile pericytes suggest these represent earlier proliferative cell states, while other cell types represent more differentiated cell phenotypes. Error bars represent standard error of the mean (SEM). (E) Trajectory analysis using Monocle algorithm of fibroblast, pericyte, muscularis and myofibroblast compartment cells highlights differentiation of pericytes and myofibroblasts from S1 (marked by *ADAMDEC1*) and S3 (marked by *OGN*) like fibroblast progenitors respectively.

Transcriptional signatures often corresponded to structural features. This was clearly seen in ECs that divided into venous, arterial, and lymphatic types with further distinction by vessel size (Figure 4A; Table S1; STAR methods). Through developmental time, we observed transition from small to large vessel ECs, reflecting intestinal angiogenesis (Figure S4A). We identified TF networks driving this such as arterial-venous differentiators *HEY1* and *SOX13* (Fawkner-Corbett et al., 2020). Both adult and fetal EC signatures were readily identifiable to vasculature in adult ST (Figure S4B).

Complimentary to ECs, a pericyte sub-type was defined by angiogenesis drivers (*PRRX1*, *THBS4*, and *ANGPT2*) (Figure 4B; Table S1; STAR methods). *ANGPT2* was a key modulator of EC-pericyte RL interactions and was confirmed to localize to the fetal vasculature (Figure S4C). Although the EC compartment was distinct with large vessel cells present at 8 PCW, pericyte populations exhibited a developmental lag. To understand these differentiation dynamics, we compared G2M and S-phase pericyte cells with G1-phase cells over developmental time, demonstrating that the majority of cells at earlier time points were highly cycling progenitors (Figure S4D; STAR methods). These populations shared transcriptional features with fibroblasts and primitive *ACTA2*^*+*^ cells, which was supported by trajectory analyses (Figure S4E). Taken together, we propose an early fibroblast-to-pericyte transition, followed by a second wave of pericyte proliferation-differentiation from immature *WNT6*-expressing cells.

Mature myofibroblast cells exhibited a similar developmental lag, appearing at 16 PCW (Figure 4Ci; Table S1). Literature suggests intestinal myofibroblasts may arise from diverse sources (McLin et al., 2009), and graph abstraction indicated mature myofibroblasts were akin to muscle cells, with distinct differences in specific genes (e.g., *RSPO2*, Figure 4Cii). Myofibroblast progenitors, however, exhibited a gradient-like spectrum with shared origins from fibroblasts and pericytes; in ST at 19 PCW, they occupied adjacent and overlapping layers to transitional stromal cells (Figures 4D and S4E).

### Enteric nervous system and muscularis propria

**Figure S5:**
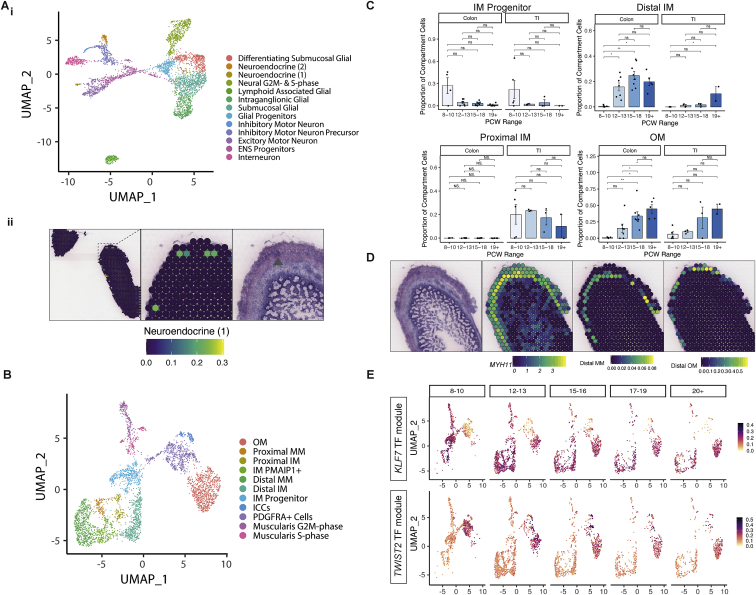
Development of muscularis and neural compartments, related to Figure 4 (A) (i) UMAP visualization of neural compartment subclusters. (ii) ST spot overlay of cell type predictions of neuroendocrine (1) cells in 19 PCW ST slide. All H&E images and corresponding ST spot overlays in (ii) are plotted over selected regions of ST H&E section A4. Full image is available in (Fawkner-Corbett et al., 2020). (B) UMAP visualization of muscularis compartment cell subclusters. (C) Selected muscle population abundance changes over developmental time course shown as bar plots. Wilcox rank test, p-value < 0.05 ^∗^; p-value < 0.01 ^∗∗^; p-value < 0.001^∗∗∗^; n.s = not significant. Error bars represent standard error of the mean (SEM). (D) ST spot overlay of *MYH11* expression (top) and MM and OM cell type predictions (bottom) localizing to distinct layers in 19 PCW colon ST slide. All H&E images and corresponding ST spot overlays in are plotted over selected regions of ST H&E section A4. Full image is available in (Fawkner-Corbett et al., 2020). (E) UMAP overlay showing muscularis-specific *KLF7* and *TWIST2* TF module AUC score distribution.

In contrast to the cell types appearing in late development, by PCW 7–8, the enteric neural crest-derived cells have migrated the length of the GI tract to pattern into submucosal and myenteric plexuses containing neurons and glial cells (Wallace and Burns, 2005). We identified distinct neuronal and glial progenitors at the earliest time point in our study (Figure 4E) and captured 5 glial cell and 7 neuron clusters (Figure S5Ai; Table S1). Alongside distinct ENS circuits highlighted by RL analysis (Figure S3C) (Fawkner-Corbett et al., 2020), this suggested relative maturity of the ENS compared to other compartments. ENS components were also clearly seen as myenteric plexuses in ST (Figure S5Aii).

In the adult intestine, neuronal plexuses are surrounded by muscle. Progenitor and differentiated intestinal smooth muscle cells (iSMCs) were respectively marked by genes such as *PLPP2* and *ACTA2* (Figure 4F; Table S1) (Fawkner-Corbett et al., 2020). We identified 11 clusters related to iSMCs including an early population of *PDGFRA*^+^ interstitial cells and interstitial cells of Cajal (Figure S5B). We observed both inner (IM) and outer (OM) muscle formation by 10 PCW. The developmental sequence of these layers in fetal intestine is unclear, with conflicting reports suggesting that both develop simultaneously or that IM develops first (Fu et al., 2004; Wallace and Burns, 2005). In line with the former, we observed a wave of differentiation, where colonic muscularis layers lagged behind more mature proximal tissue—early samples were dominated by progenitor populations, whereas differentiated distal muscularis mucosa (MM), OM, and IM cells largely appeared after 12 PCW (Figure S5C). In ST of the colon at 19 PCW, but not 12 PCW, the muscle layers were separated and visually distinct (Figure S5D).

We identified key TF networks delineating muscularis cells; *KLF7* in muscle and *TWIST2* in interstitial and OM cells (Figure S5E). *FOXF2* was active specifically in IM (Figure 4F), similar to reports in mice (Bolte et al., 2015). Furthermore, it’s reported that *Foxf2*^−/−^ mice are embryonically lethal with a thin-walled colon (Ormestad et al., 2006), reduced number of enteric neurons, a flat epithelium, and undeveloped fibroblasts. This finding aligns with the possibility that smooth muscle-mediated mechanical forces are responsible for initiating villus formation.

### Mesenchymal cells of the developing intestinal lamina propria

**Figure S6:**
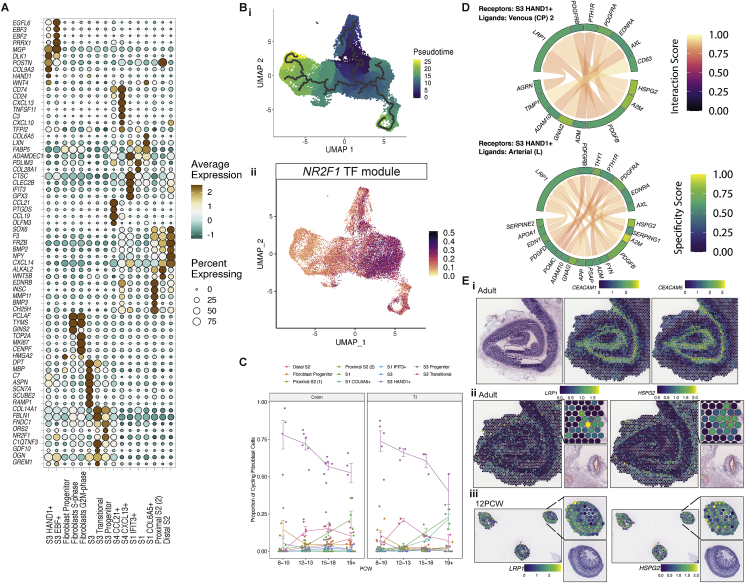
Development of fibroblast compartment, related to Figure 4 (A) Dot plot heatmap showing selected fibroblast cell sub cluster markers. Points are scaled by percentage of cells with at least minimal (>0) detection of marker within the cluster and colored by mean cluster expression. (B) Developmental trajectory analyses of fibroblast compartment cells using Monocle algorithm shown over UMAP embedding, colored by pseudotime (left). UMAP overlay (right) of *NR2F1* TF module AUC scores, delineating S1/S2 type cells from S3 type fibroblasts. (C) Time course changes in the proliferating S- and G2M- phase cells in the fibroblast compartment. S3 progenitor population constitutes the most abundant and the most enriched (data not shown) over G1-phase cells population. Error bars represent standard error of the mean (SEM). (D) Circos plot visualizing putative cross-talk between S3+ HAND1+ cells and Arterial and Venous endothelial cells. (E) ST adult slide spot overlay of expression of receptor-ligand pair *CEACAM1* and *CEACAM5*, showing significant co-localization of these molecules (i). ST slide overlay of *LRP1* and *HSPG2* receptor-ligand pair in adult ST slide (ii) and 12 PCW ST slide (iii). All H&E images and corresponding ST spot overlays in are plotted over selected regions of ST H&E sections A1 (i-ii) and A6 (iii), reference H&E images repeated for clarity. Full images are available in (Fawkner-Corbett et al., 2020).

As the intestine develops, these diverse compartments are supported by mesenchymal cells, the largest compartment within our atlas (24,081 cells). We designated our fibroblast cells stromal 1–4 (S1–S4) subtypes, based on the nomenclature established in adult scRNA-seq (Kinchen et al., 2018). Temporal, locational, and cycling differences further sub-divided these into 16 clusters (Figure 4G; Table S1; STAR methods). 3 clusters of S1 cells formed the bulk of submucosal structural cells, whereas S2 cells (marked by *F3*, *NPY*, and *FOXL1*) expressed markers akin to a peri-cryptal telocyte population (Degirmenci et al., 2018; Shoshkes-Carmel et al., 2018) that is important in epithelial development and support (Kaestner et al., 1997; McCarthy et al., 2020) (Figure S6A) (Fawkner-Corbett et al., 2020).

In contrast to adult, not only did S3-like cells comprise a major fibroblast population *in utero*, they also exhibited greater heterogeneity. Some S3 clusters had high expression of primitive markers (*HAND1*, *WNT4*, and *DLK1*), alongside adult hallmark S3 genes (*C7* and *CCDC80*) (Figure S6A). Trajectory analysis identified a branch point where immature S3-like cells divided into S3 or S1/2/4 lineages that could be differentiated by lineage-specific TF networks, such as NR2F1 (Figure S6B) (Fawkner-Corbett et al., 2020). *NR2F1* was also expressed in myofibroblast cells in line with our shared trajectory analysis (Figure S4E) (Fawkner-Corbett et al., 2020). We identified the S3-like progenitor population to be highly enriched in the proliferative compartment, suggesting these cells may give rise to the bulk of differentiated fibroblast populations in this period (Figure S6C).

To better understand S3 subtype function, we studied RL interactions, noting a large number of cross-talk predictions with ECs (Figure S6D). A number of these were mediated through the receptor *LRP1* that regulates tissue plasminogen activation in colonic fibroblasts (Hardy et al., 1997). To confirm this in ST data, we carried out RL spatial co-expression analysis (STAR methods), which identified strongly spatially co-localizing RL pairs such as *CEACAM1* and *CEACAM5* toward the crypt top (Figure S6Ei). We observed significant co-localization of *LRP1* with the ligand *HSPG2*, a key component of vascular extracellular matrix, in adult and fetal tissue (p = 2.17 × 10^−112^ radius of spots and p = 2.37 × 10^−29^ in same spot adult; p = 2.9 × 10^−19^ radius and p = 0.005 same spot 12 PCW) (Figures S6Eii and S6Eiii).

In fetal ST slides, we localized S3 clusters to depths and areas related to EC populations (Figure 4Hi). We localized expression of several S3-specific genes, such as *C7*, to spots adjacent to vasculature structures in adult tissue, as these structures were more clearly visible than in fetal sections (Figure 4Hii). Transferring fetal cell-type labels to adult ST slides, we show adult counterparts of S3 HAND1^+^ and S3 EBF^+^ cells congregate around large vessels (Figures 4Hiii–4Hiv), recapitulating adult S3. This highlights a possible role for these cells as forming a supportive niche for enteric vessels. This was confirmed by pairwise cell-type signal correlation analysis (STAR methods), where ECs, pericytes, and “S3” type fibroblast signals correlated within the same ST spots (Figure 2Ci). Thus, our atlas demonstrates the strength of studying compartments in tandem to better understand compartmental crosstalk required for normal development.

### Unbiased mapping of morphogen gradients directing intestinal development

**Figure 5:**
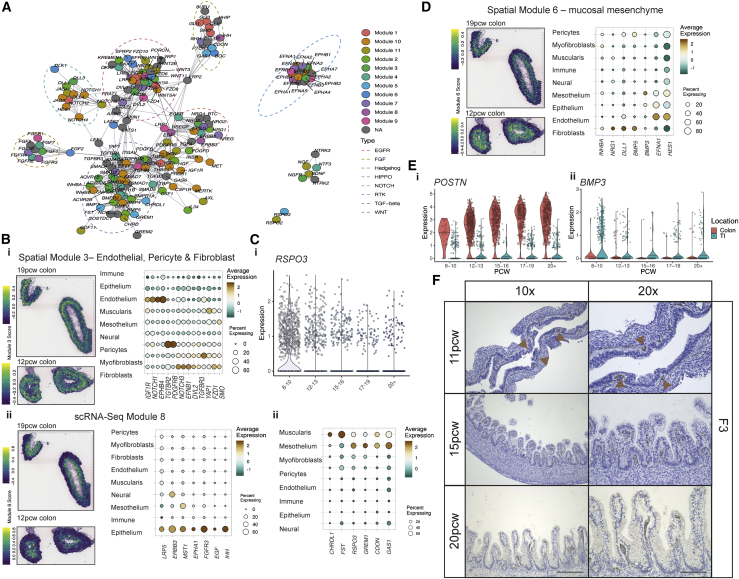
Intestinal morphogen gradients in specific cell types and spatial locations (A) Graph visualization of morphogen molecule STRING interactome. Communities enriched for EGFR, FGF, Hedgehog, HIPPO, NOTCH, RTK, TGF-beta and WNT signaling pathways are highlighted in dashed ellipses. Nodes are colored by scRNA-Seq module. Nodes unassigned to a module are shown in grey (NA). (B) Individual morphogen module overview shown as a module score overlay in ST spots in slides at 19 and 12 PCW colon and a dotplot showing module gene expression at compartment level for ST module 3 which captures endothelial, pericyte and fibroblast morphogens (i) and scRNA-Seq module 8, which captures epithelial morphogens (ii). All spot overlays shown are plotted over ST H&E sections A4 (top) and A3 (bottom, rotated). Full images are available in (Fawkner-Corbett et al., 2020). (C) (i) Violin plot showing expression of *RSPO3* over developmental time course in scRNA-Seq of muscularis compartment cells. (ii) Dotplot showing the expression of mesothelium and muscularis-specific morphogen scRNA-Seq module 5 genes (D) Individual morphogen module overview shown as module score overlay in ST spots in slides at 19 and 12 PCW colon and a doplot showing expression of fibroblast-specific morphogen spatial module 6. All spot overlays are plotted over ST H&E sections A4 (top) and A3 (bottom, rotated). Full images are available in (Fawkner-Corbett et al., 2020). (E) Violin plots depicting selected S2 genes (*POSTN* (i) and *BMP3* (ii)) that show locational and time-course differences in expression. (F) Representative images of colonic sections from 11, 15 and 20 PCW embryonic tissue stained for F3 protein by IHC. Brown arrows within the 11 PCW sample indicate positive F3 staining below newly developing invaginations/hillocks in epithelium (*n* = 3 for each individual image with +-1pcw samples, 10x/20x magnification scale bar=360/180 μm)

**Figure S7:**
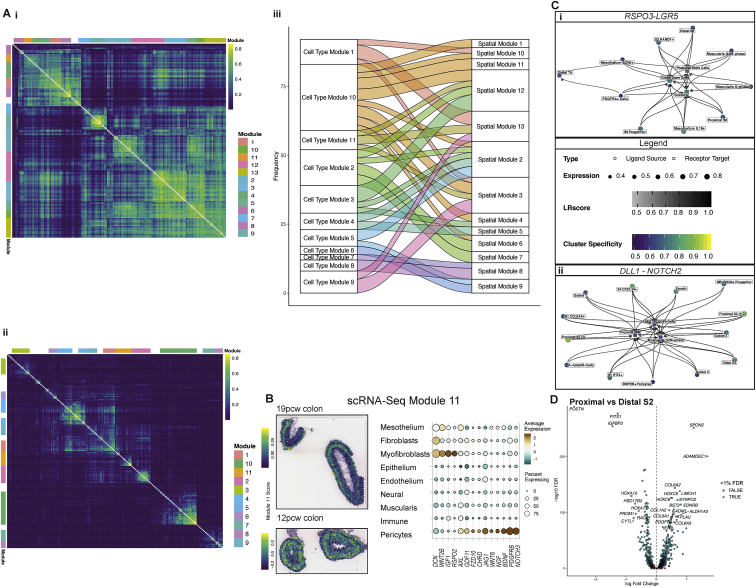
Cell-type specific and spatial morphogen gradients in the developing intestine, related to Figure 5 (A) Heatmap visualizing the correlation structure of morphogen module genes expressed in ST data (i) and single cell RNA-Seq data (ii). The overlap between detected modules is visualized in (iii). (B) Individual morphogen module overview shown as a module score overlay in ST spots in slides at 19 and 12 PCW colon and a dotplot showing module gene expression at compartment level for scRNA-Seq module 11, which captures largely pericyte and myofibroblast-derived morphogens. Spot overlays are plotted over ST H&E sections A4 (top) and A3 (bottom, rotated). Full images are available in (Fawkner-Corbett et al., 2020). (C) Interacting cell network plots showing putative cell type cross talk via specific receptor-ligand pairs, *RSPO3/LGR5*(i) and *DLL1-NOTCH2*(ii). Ligand source clusters are indicated as circles, receptor target cells as squares; autocrine interactions are shown as diamonds. Edges color indicates interaction score, node color ligand or receptor cell type specificity and node size indicates percentage of cells expressing ligand or receptor in the cluster. (D) Volcano plot visualizing differentially expressed genes between colonic and TI S2 populations.

A fundamental question in understanding intestinal development is how local morphogen gradients and their antagonists shape intestinal villus morphogenesis (Beumer and Clevers, 2020). We created a morphogen map of genes involved in 8 pathways to clarify where these molecules act in space and time (STAR methods) (Fawkner-Corbett et al., 2020). Co-expression analyses identified 11 cell-type-specific and 13 spatially co-localizing morphogen modules, which we scored in all ST slides for module activity (Figures 5A and S7A) (Fawkner-Corbett et al., 2020). These modules often aligned with tissue depth in ST. Spatial module 3 consisted of morphogens from ECs, fibroblast and pericyte origin, including *LRP1* and was deep in tissue (Figure 5Bi). Epithelial specific modules expressed genes from Hedgehog pathway (*IHH*), e.g. the Frizzled co-receptor *LRP5* that is important in Wnt signaling (Fenderico et al., 2019), and localized near the lumen (Figure 5Bii). Another module containing myofibroblast components, with the morphogen *WNT2B* and receptor *RSPO2*, appeared diffusely at 12 PCW and became localized later in time (Figure S7B). This agreed with the finding that mature myofibroblasts appeared after epithelial components, suggesting the ISC-myofibroblast signaling circuit is not established until after crypt formation. *WNT2B* was expressed by mesothelium, providing the only source of the ligand prior to myofibroblast differentiation (Figure S7B). Similarly, *RSPO3*, which can signal to ISCs via *LGR5* (Figure S7Ci), was high in a mesothelium/muscularis module and seen predominantly before 12 PCW and then lost over time as tissue depth increased (Figure 5C). This suggests a paradigm where, as the developing intestine grows, morphogen gradients that may be otherwise broken by increasing physical distance between layers could be restored via expression from sequentially developing cell types.

### S2 fibroblasts are location-specific and control epithelial patterning

Peri-cryptal S2 fibroblasts or telocytes (McCarthy et al., 2020) provide epithelial support through expression of ligands from the transforming growth factor β (TGF-β) superfamily and WNT pathways. We identified three distinct S2 clusters in development expressing key morphogens from both TGF-β (*BMP2*, *BMP4*, and *BMP5*) and non-canonical WNT pathways (*WNT5A* and *WNT5B*) (Figure S6A) (Fawkner-Corbett et al., 2020). These S2-specific genes formed part of a predominantly submucosal fibroblast morphogen module (scRNA-seq module 2) that encompassed *DLL1*, *BMP5*, and *NRG1* (Figure 5D). Together with RL interactions, such as *DLL1*-*NOTCH2* (Figure S7Cii), this highlighted S2 cells and the morphogen-rich niche they provide as important in epithelium formation.

Unlike other fibroblast populations, TI and colonic S2 cells were surprisingly different with 885 differentially expressed genes (<5% FDR) (Figure S7D) (Fawkner-Corbett et al., 2020) including colon-specific expression of *POSTN* or TI-prominent *PDGFRA* (Figure S7D). Many location-specific differences were detected before 10 PCW, representing a strong locational identity before crypts/villi form—for instance *POSTN* and *BMP3* were seen very early within their respective S2 sub-types (Figure 5E). Such key locational differences in S2 morphogen profiles suggest a mechanism by which vastly different epithelial morphologies can develop.

Finally, we confirmed the S2 marker *F3* was present before crypt formation and formed hillocks that increased upward as villi formed (Figure 5F). Thus, in human intestine, S2 type fibroblasts are required not only for epithelial crypt-niche maintenance but may also play an active role in its formation.

### Development of human intestinal immunity

#### Immune cell heterogeneity during lymphoid tissue formation

**Figure 6:**
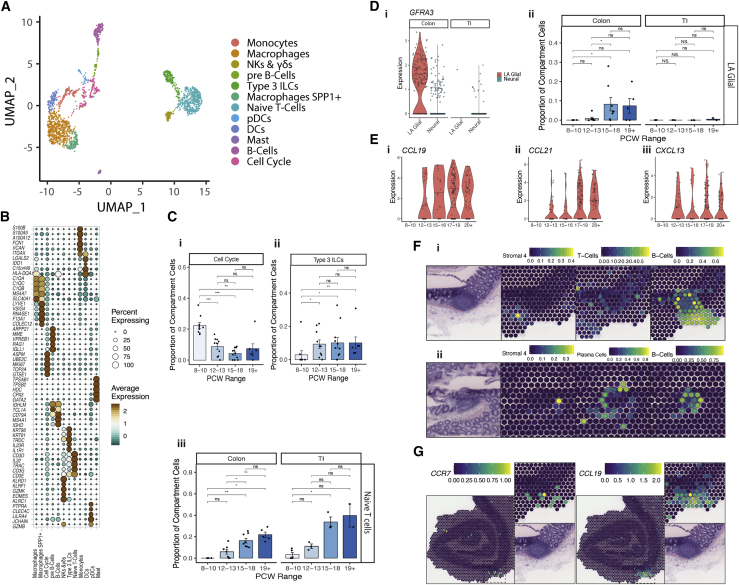
Early intestine immune colonization and immune-stromal interactions (A) UMAP visualization of immune compartment cell clusters. (B) Dot plot showing the expression of key immune compartment cluster markers. (C) Barplots showing time course changes in selected immune compartment cluster abundance. Wilcox rank test, p-value < 0.05 ^∗^; p-value < 0.01 ^∗∗^; p-value < 0.001^∗∗∗^; ns = not significant. Error bars represent standard error of the mean (SEM). (D) (i) Violin plot showing expression of GFRA3 in lymphoid associated glial (LA Glial) cells and other neural cells in cells from colon and TI. (ii) Barplot showing time course changes in LA Glial abundance in neural compartment in colon and TI. Wilcox rank test, p-value < 0.05 ^∗^; p-value < 0.01 ^∗∗^; p-value < 0.001^∗∗∗^; ns = not significant. Error bars represent standard error of the mean (SEM). (E) Violin plots showing expression of *CCL19* (i), *CCL21* (ii) and *CXCL13* (iii) in S4 fibroblast cells over developmental time course. (F) ST cell type predictions in spots overlaying a submucosal lymphoid aggregate in adult tissue slide showing scores for adult Stromal 4, T-cells, B-cells and Plasma cells in the bottom of slide A1 (i) and center of slide A2(ii). H&E images and spot overlays show selected regions from ST sections A1 (i) and A2 (ii). Full images are available in (Fawkner-Corbett et al., 2020). (G) Expression of *CCR7* and *CCL19* in ST adult slides. H&E images and all spot overlays shown are plotted over selected regions of ST H&E section A1, H&E image of zoomed in section repeated for clarity. Full image is available in (Fawkner-Corbett et al., 2020).

**Figure S8:**
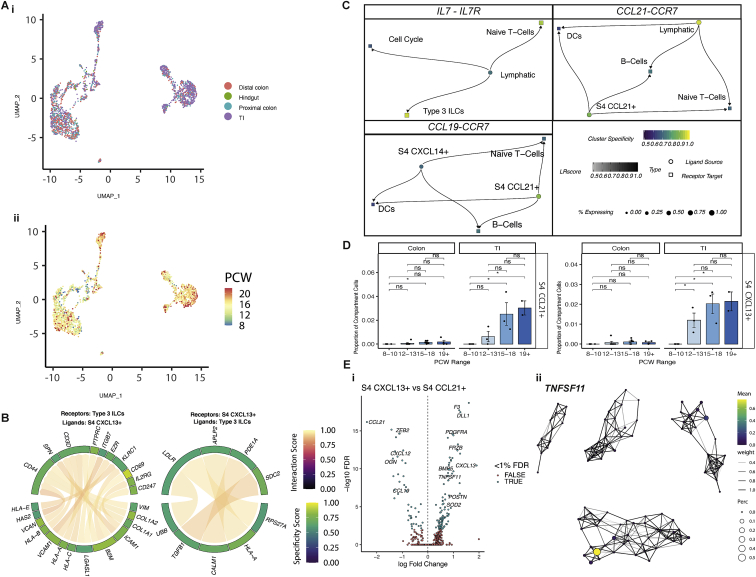
Immune colonization of developing intestine, related to Figure 6 (A) UMAP visualization of immune compartment cell cluster locational (i) and PCW (ii) distributions. (B) Circos plot visualizing putative cross-talk between ILC3s and S4 CXCL13+ cells. (C) Interacting cell network plots showing cell type cross talk via specific receptor-ligand pairs, *IL7/IL7R, CCL21/CCR7* and *CCL19/CCR7*. Ligand source clusters are indicated as circles, receptor target cells as squares; autocrine interactions are shown as diamonds. Edges color indicates interaction score, node color ligand or receptor cell type specificity and node size indicates percentage of cells expressing ligand or receptor in the cluster. (D) S4 cell sub-population (left- S4 CCL21+; left- S4 CXCL13+) abundance changes over developmental time course in colon and TI samples shown as bar plots. Wilcox rank test, p-value < 0.05 ^∗^; p-value < 0.01 ^∗∗^; p-value < 0.001^∗∗∗^; n.s = not significant. Error bars represent standard error of the mean (SEM). (E) Volcano plot (i) visualizing differentially expressed genes between S4 CXCL13+ and S4 CCL21+ cell subpopulations. Global expression of *TNFSF11* (*RANKL*) is visualized as a graph abstraction overlay in (ii).

Our atlas captured 2,199 immune cells across development (Figures 6A and 6B; Table S1; STAR methods) emerging from 6 lineages (macrophage, monocyte, dendritic cell, eosinophils, adaptive, and innate lymphoid cells). Immune cells were more prevalent in SI (mean, 3%–8% of all cells captured) than colon (mean, 1%–2.5%) and overall were particularly rare before the first trimester. Before 10 PCW, we observed an enrichment of myeloid cells (Figure S8A), whereas at 12 PCW, there was an influx of naive CD4^+^ and CD8^+^ T cells, natural killer (NK), type 1 innate lymphoid cells (ILCs), and type 3 ILCs (Figure 6C). The latter were substantially more prevalent in fetal intestine than adult (Martin et al., 2019), accounting for up to 30% of all captured immune cells in some late samples.

Fetal ILC3s expressed *IL7RA* and *ID2* (Table S1), which is vital for *in utero* Peyer’s patch (PPs) formation in mice, thus distinguishing these cells as lymphoid tissue inducer (LTi) type 3 ILCs (Boos et al., 2007; van de Pavert and Mebius, 2010). PP formation is a coordinated process with interaction between LTi cells, ECs, and stromal cells. Furthermore, murine studies show a neural *RET*/*ARTN*/*GFRA3* axis is also required, with *GFRA3*-deficiency causing impaired PP development (Veiga-Fernandes et al., 2007). The formation of gut-associated lymphoid tissue (GALT) is thought to occur prenatally in the SI as PPs and postnatally in the colon (van de Pavert and Mebius, 2010). Conversely, here, we identified a unique colonic rather than SI-specific glial population serving as the sole *GFRA3* source during development and expanding at 15 PCW (Figure 6D), suggesting similar neural-immune circuits may exist during human colon development.

#### S4 fibroblasts are a keystone in lymphoid structure formation and maintenance

Other key mediators of PP formation include stromal organizer cells that facilitate LTi tissue homing via CCL19, CCL21, and CXCL13 (van de Pavert and Mebius, 2010). We found these localized specifically to the two S4 fibroblast clusters, and expression increased with development (Figures 6B and 6E). Mapping RL interactions in S4 clusters highlighted cross-talk with diverse immune cells, including ILC3 signaling via *VCAM1*-*ITGB7* (Figure S8B) (Fawkner-Corbett et al., 2020). This further supports a role for S4 in lymphoid tissue formation—these adhesion molecules are required in GALT formation (Yoshida et al., 2001). LTi cells and S4 cells also showed interactions via *IL7* and *CCL19*/*21* with their receptors *IL7R*/*CCR7* (Figure S8C), which, if absent in mice, lead to failure to form secondary lymphoid follicles (Ohl et al., 2003).

To confirm these interactions spatially, we studied adult ST slides where submucosal lymphoid follicles were seen as distinctive structures. Key marker genes of S4 and immune cells (e.g., *CD3* and *CD19*) were expressed in and around these follicles, and factor analysis confirmed various adult immune (B cells, T cells, and myeloid cells) and S4 cell types localized here (Figure 6F). We also found significant co-localization of key RL pairs in ST, including *CCR7*/*CC19* (p = 1.098 × 10^−10^ within 1 spot, p = 5.59 × 10^−49^ within radius) (Figure 6G). Thus, we show that S4 cells are immune follicle-adjacent fibroblasts in adult colon that appear in a time-dependent manner during fetal GALT development.

Both S4 clusters were largely SI-specific, supporting *in utero* development of PPs but not colonic GALT, and were entirely absent pre-12 PCW (Figure S8D). Comparing the transcriptional profiles of these two populations, we found that S4 CXCL13^+^ subtype exhibited many of the key signatures of peri-cryptal S2 fibroblasts (*POSTN*, *F3*, *PDGFRA*, and *BMP5*) and were the only intestinal source of *RANKL*/*TNFSF11* (Figure S8E)—deficiency of which results in impaired PP formation and is required for differentiation of M cells in PPs. Taken together, this suggests the same peri-cryptal fibroblasts in PPs are acting as the epithelial crypt-niche support cells, coordinators of lymphoid tissue formation, and mediators of stromal-immune cross-talk, thus revealing their hitherto unappreciated highly dynamic roles.

### Charting the cellular basis of congenital intestinal disorders

The pathogenesis of congenital intestinal diseases remains poorly defined, because underpinning genetic defects are rare, and some abnormalities occur early *in utero*. Intestinal ventral herniation, elongation, rotation, and repositioning all occur in the first trimester, and deviation can lead to defects such as omphalocele (Adams and Stanton, 2014).

**Figure 7:**
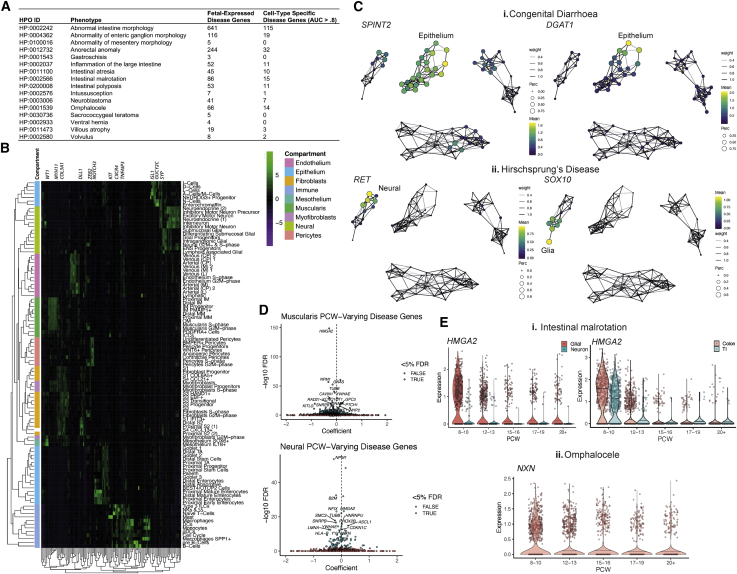
Application of *in utero* gene expression profiles to developmental disease (A) Table summarizing intestinal disease HPO phenotype terms and the number of associated genes which are at least minimally expressed in scRNA-Seq dataset or highly cell type specific. (B) Heatmap visualizing mean scaled cluster expression of cell-type specific disease genes summarized in (A). (C) Graph abstraction overlay of cluster expression of disease genes associated with (i) congenital diarrhea (*SPINT2* & *DGAT1)* and (ii) Hirschsprung’s Disease (*RET* & *SOX10*). (D) Volcano plots highlighting top time-course varying disease-associated genes in muscularis and neural compartments. (E) Violin plots showing individual time-course varying disease-associated genes in (i) intestinal malrotation, showing *HMGA2* expression over time in glial vs neuron cells (left) and *HMGA2* expression over time in muscularis compartment in TI and colon (right); and (ii) omphalocele showing *NXN* expression over time in neurons.

To reveal time-critical transcriptional defects that might drive congenital intestinal diseases, we correlated our data with a curated list of perinatal intestinal diseases from the Human Phenotype Ontology (HPO) annotated with hereditary phenotypes (STAR methods) (Fawkner-Corbett et al., 2020). By integrating 749 known disease genes with our scRNA-seq data, we linked congenital disorders with phenotypes that likely manifest through highly cell-type-specific defects (Figures 7A and 7B) and result in disorders of intestinal, ventral, perineal, aganglionic, inflammatory, or oncological pathology (Figure 7A, (Fawkner-Corbett et al., 2020).

These encompassed pan-epithelial *SPINT2* and *DGAT1* in distal enterocytes (area under the curve [AUC] = 0.80), where defects can result in forms of severe congenital diarrhea requiring parenteral nutrition (Haas et al., 2012; Holt-Danborg et al., 2019) (Figure 7Ci). *SOX10* and *RET* are associated with Hirschsprung’s disease (intestinal aganglionosis) (Heuckeroth, 2018) and were expressed in ENS (Figure 7Cii), including lymphoid-associated glial (AUC = 0.814) cells. Given that these cells play a role in lymphoid follicle formation (Veiga-Fernandes et al., 2007), our data suggest a potential link to a complication of Hirschsprung’s disease—enterocolitis—that originates through complex neuro-immune interplays after aganglionic intestine is removed (Heuckeroth, 2018).

The temporal aspect of our data allowed study of disease genes that varied over developmental time (Figure 7D) (Fawkner-Corbett et al., 2020) and could provide insight to time-linked conditions. We highlight *HMGA2*, a pre-12 PCW glial and IM progenitor-expressed gene (FDR <2.2e−16, respectively) (Figure 7Ei), linked with intestinal malrotation (Mari et al., 2009) (12q14 microdeletion syndrome, ORPHA: 94063). Similarly, pathogenic variants of early inhibitory motor neuron-specific *NXN* (Figure 7Eii) can lead to omphalocele (White et al., 2018) (Robinow syndrome, OMIM: 618529). Because at this time of development, the intestine returns to the abdomen, our results highlight that ENS cells and muscular progenitors could be vital for this process.

## Discussion

Recent scRNA-seq studies have identified diversity in cell types and states within epithelial, mesenchymal, and immune cells of the adult intestine (Kinchen et al., 2018; Martin et al., 2019; Parikh et al., 2019; Smillie et al., 2019). In many cases, their origins, role, and timing in development remain unclear; however, ongoing work is beginning to address this using unbiased, high-throughput methods (Elmentaite et al., 2020; Holloway et al., 2020) and expanding on earlier smaller scale studies (Gao et al., 2018).

A key advantage of our study lies in the capture of full thickness intestinal tissue rather than biopsy samples, enabling us to map all intestinal compartments. Our data also cover several critical developmental events—epithelial crypt-villus formation, differentiation of the mesenchyme, establishment of muscle layers, expansion of vascular systems, immune colonization, and the emergence of GALT. Thus, we provide insights into the coordinated emergence of different populations, define TF networks regulating their development, and chart the nature of their cross-talk with neighboring cells.

We identified programs that differentiate SI and colonic epithelium, revealing that locational cell fate decisions leading to the distinctive cellular anatomy of mature small and large intestine are made prior to crypt-villus formation. We observed a predominance of progenitor stem-like cells in early development, with formation of *LGR5*^+^ hillocks at 12 PCW. In sharp contrast to most other epithelial cells, BEST4/OTOP2 cells transcriptional signature was already established prior to crypt formation and independent from the crypt-villus axis dynamics observed at later time points. Although the types of epithelial cells observed at 22 PCW were similar to adult tissue, the proportion of immature cells was higher. This prevalence of immature epithelia may contribute to diseases such as necrotizing enterocolitis in premature infants (Fitzgibbons et al., 2009; Ravisankar et al., 2018).

Recent work established that gut fibroblasts are diverse in their transcriptional and functional attributes (Kinchen et al., 2018; Martin et al., 2019; Smillie et al., 2019). We show that telocytes/S2 fibroblasts, located in proximity to intestinal epithelium, are a key source of morphogens required for epithelial crypt support and emerge prior to crypt formation localizing to “hillocks” as the epithelium begins to warp. These peri-cryptal cells were highly distinct between the SI and colon. Similarly, we establish a function for the “S3” type fibroblasts as “vasculature-niche cells,” because ST localized these cells congregating around large vessels. These cells expressed complement components, suggesting involvement in local tissue remodeling during vessel development.

The temporal dynamics of cell subtypes shed light on morphogenesis, demonstrating some populations emerge in tandem, whereas others create niches paving the way for differentiation or colonization by lagging populations. This is evident in GALT formation, where the coordinated emergence of LTi and S4 populations at 12 PCW was followed by increasing B and T cell emergence (Figures 6C and 6E). S4 fibroblasts were first reported as ulcerative colitis (UC)-associated cells, bearing features of follicular reticular cells (Kinchen et al., 2018). Here, we definitively localize them to submucosal lymphoid aggregates in adult colon using ST and further define two fetal sub-populations that may guide formation of GALT and PPs *in utero*.

We harness the strength of these data to provide insights into neonatal disease. We tracked genes linked to these genetic defects to highly specific time points and cell types, thus revealing information about diseases that are challenging to study *in utero*.

Last, we present all aspects of these highly dimensional data via an interactive resource, spatio-temporal analysis resource of fetal intestinal development (STAR-FINDer).

## STAR★methods

### Key Resources Table

**Table:** 

REAGENT or RESOURCE	SOURCE	IDENTIFIER
**Antibodies**		

Transferrin mouse anti human antibody	R&D	Cat# AF3987, RRID:AB_10890212
BEST4 rabbit anti human antibody	Sigma	Cat# HPA058564, RRID:AB_2683759
ANGPT2 goat anti human antibody	R&D	Cat# AF623, RRID:AB_355483
F3 rabbit anti human antibody	Sigma	Cat# HPA049292, RRID:AB_2680701
Hs-LGR5	ACDBio	CAT# 311021
GATA4 rabbit anti human antibody	AbCAM	Cat#Ab124265, RRID: AB_11000793
DAPI solution	BD Pharminogen	Cat#564907
CD90(Thy1) FITC anti-human	Biolegend	Cat#328107 RRID:AB_893438
CD326(EpCAM) PE-vio770	Milteyni Biotec	Cat# 130-099-742 RRID:AB_2660305
CD45 APC human	Milteyni Biotec	Cat# 130-098-143 RRID:AB_2660416
CD326(EpCAM) (used for conjugation of hash tag oligonucleotide)	Biolegend	Cat# 324229, RRID:AB_2563742
Totalseq A0251 anti-human hashtag 1	Biolegend	Cat# 394601, RRID:AB_2750015
Totalseq A0252 anti-human hashtag 2	Biolegend	Cat# 394603, RRID:AB_2750016
Totalseq A0253 anti-human hashtag 3	Biolegend	Cat# 394605, RRID:AB_2750017
Totalseq A0254 anti-human hashtag 4	Biolegend	Cat# 394607, RRID:AB_2750018
Totalseq A0255 anti-human hashtag 5	Biolegend	Cat# 394609, RRID:AB_2750019
Totalseq A0256 anti-human hashtag 6	Biolegend	Cat# 394611, RRID:AB_2750020
Totalseq A0257 anti-human hashtag 7	Biolegend	Cat# 394613, RRID:AB_2750021
Totalseq A0258 anti-human hashtag 8	Biolegend	Cat# 394615, RRID:AB_2750022
Totalseq A0259 anti-human hashtag 9	Biolegend	Cat# 394617, RRID:AB_2750023

**Biological Samples**		

Human adult colon resections	John Radcliffe NHS Foundation trust. (REC reference: 18/WM/0237)	REC reference: 18/WM/0237Sample overview detailed in Supplemental Data (Fawkner-Corbett et al., 2020)
Fetal Intestinal Samples	Human Developmental Biology Resource (HDBR), London	HBDR project 200462, REC: 18/LO/0822. Sample Overview detailed in Supplemental Data (Fawkner-Corbett et al., 2020)

**Chemicals, Peptides, and Recombinant Proteins**		

L-15 (Lebowitz) media	Sigma	Cat#L1518
Phosphate Buffered Saline (PBS)	Oxoid Ltd or Sigma (experiment dependent)	Cat#BR0014G / D8537-500ML
CryostorCS10	Sigma	Cat#C2874-100ML
OCT Embedding matrix for frozen sections	CellPath	Cat#KMA-0100-00A
Ispoentane (2-Methylbutane)	Sigma	Cat#277258-1L
RPMI-1640 medium	Sigma	Cat#R8758-500ml
Dulbesco’s Modified Eagle’s Medium (DMEM)	Sigma	Cat#D5796-500ML
Penicillin-Streptomycin	Sigma	Cat#P0781-100ML
HEPES Buffer Solution (1M)	GIBCO	Cat#15630-056
Fetal Calf Serum / Fetal Bovine Serum	Sigma	Cat#F9665-500ML
Ultrapure 0.5M EDTA, ph8.0	invitrogen	Cat#15575-038
Tryple Express	GIBCO	Cat#12605-028
Deoxyribonuclease I from bovine pancreas (DNase)	Sigma	Cat#DN25-10mg
Umbilical Cord Dissosciation Kit, Human	Milteyni Biotec	Cat#130-105-737
Bovine Serum Albumin	Sigma	Cat#A7906-100G
Hydrogen Peroxide 30%	Merck	Cat#1.07210.1000
Hematoxylin QS (used for IHC)	Vector	Cat#H-3404
Mayer’s Hematoxylin (used for ST)	Dako	Cat#S3309
Dako Bluing Buffer (used for ST)	Dako	Cat#CS702
HBSS medium	Lonza	Cat#10-543F
Eosin Y solution	Sigma	Cat#HT110216-500ml
Borate Buffered Saline (BBS)	Sigma	Cat#08059-100-TAB-F
TCO(Trans-Cyclooctene)-PEG4-NHS Click chemistry in DMSO	Biomers Ltd	Custom made as per (Stoeckius and Hao,). https://www.biomers.net

**Critical Commercial Assays**		

10x Chromium Single Cell 3′ GEM, Library & Gel Bead Kit v3	10x Genomics	Cat#1000075
High sensitivity DNA reagents (used with Agilent Bioanalyzer 2100 system)	Agilent Technologies	Cat#5067-4626
QuBit dsDNA HS Assay Kit (used with QuBit 3.0)	invitrogen	Cat#Q32851
Novaseq 6000 S4 150bp PE reads	Illumina	Cat#20012866
Nextseq 500/550 Hi Output kit v2.5	Illumina	Cat# 20024907
Visium Spatial Gene Expression Slide & Reagent Kit	10x Genomics	Cat# 1000187
KAPA SyBR FAST qPCR kit	KAPA biosystems	Cat # KK4600
KAPA library quant kit (illumina) universal qPCR mix	Kapa biosystems	Cat# KK4824
Immpress HRP Reagent kit (peroxidase)	Vector	Species specific (Mouse Cat#MP7452, rabbit Cat#MP7451, goat Cat#MP74050)
Immpact DAB Peroxidase substrate	Vector	Cat# SK-4105
RNAScope 2.5HD assay brown	ACDBio	Cat# 322310
Agilent RNA 6000 Pico Reagents (used with Agilent 2100 bioanalyzer system)	Agilent Technologies	Cat# 5067-1513

**Deposited Data**		

Single cell RNA-Seq data generated in this study	http://www.ncbi.nlm.nih.gov/geo	GSE158702
Spatial transcriptomics data generated in this study	http://www.ncbi.nlm.nih.gov/geo	GSE158328
Adult scRNA-Seq reference dataset used in this study	http://www.ncbi.nlm.nih.gov/geo	GSE114374
Adult scRNA-Seq reference dataset used in this study	http://www.ncbi.nlm.nih.gov/geo	GSE116222
Supplemental data used in this study	Mendeley Data: “Spatiotemporal Analysis of Human Intestinal Development at Single Cell Resolution: Supplementary Data”	https://doi.org/10.17632/gncg57p5x9.2

**Oligonucleotides**		

Hash tag oligonucleotide primer / ADT additive primer/ SI-PCR primer	Sigma oligo store	See (Stoeckius et al., 2018; Stoeckius and Hao., 2020), and supplemental data (Fawkner-Corbett et al., 2020) for full sequences
Custom oligonucleotide for modification of HTO library preparation	Sigma oligo store	“10x genomics PCR primer”; “ADT cDNA PCR additive primer”; “Truseq DS_1”; “Truseq DS_2”; “Truseq DS_3”; “Truseq DS_4”; “Truseq DS_5”; “Truseq DS_6.” Full sequences listed in supplemental data (Fawkner-Corbett et al., 2020 – Tab 22) and applied with step by step protocol (Stoeckius and Hao., 2020 - https://citeseq.files.wordpress.com/2019/02/cite-seq_190213.pdf)

**Software and Algorithms**		

FiJi v2.0.0	ImageJ	https://imagej.nih.gov/ij/
QuantStudio 7-flex PCR software	Thermofisher	https://www.thermofisher.com/us/en/home/global/forms/life-science/quantstudio-6-7-flex-software.html
FlowJo v10.7.1	Flowjo	https://Flowjo.com
Illumina bcl2fastq version 2.20.0.422	Illumina	https://Illumina.com
fastQC version 0.11.9	Babraham Institute	https://www.bioinformatics.babraham.ac.uk/projects/fastqc/
Cellranger version 3.1.0	10x Genomics	https://support.10xgenomics.com/single-cell-gene-expression/software/downloads/latest
Spaceranger version 1.0.0	10x Genomics	https://support.10xgenomics.com/single-cell-gene-expression/software/downloads/latest
CITE-seq Count version 1.4.3	Github	https://github.com/Hoohm/CITE-seq-Count
R package DropletUtils version 1.4.2	R Bioconductor; Lun et al., 2019	https://www.bioconductor.org/packages/release/bioc/html/DropletUtils.html
R package Seurat version 3.1.5.9900	Github; Butler et al., 2018	https://github.com/satijalab/seurat
R package Harmony version 1.0	Github; Korsunsky et al., 2019	https://github.com/immunogenomics/harmony
R package MAST version 1.14.0	R Bioconductor; Finak et al., 2015	https://www.bioconductor.org/packages/release/bioc/html/MAST.html
R package zinbwave version 1.10.1	R Bioconductor; Risso et al., 2018	http://bioconductor.org/packages/release/bioc/html/zinbwave.html
SCENIC	Github; Aibar et al., 2017	https://github.com/aertslab/SCENIC
R package SeuratWrappers version 0.1.0	Github	https://github.com/satijalab/seurat-wrappers
R Package SingleCellSignalR version 1.0	R Bioconductor; Cabello-Aguilar et al., 2020	http://www.bioconductor.org/packages/release/bioc/html/SingleCellSignalR.html
R package WGCNA version 1.69	R CRAN; Langfelder and Horvath, 2008	https://cran.r-project.org/web/packages/WGCNA/index.html
STRING Database version 11.0	Szklarczyk et al., 2019; STRING Database	https://string-db.org/
R package clusterProfiler version 3.16.1	R Bioconductor; Yu et al., 2012	https://bioconductor.org/packages/release/bioc/html/clusterProfiler.html
R package ggplot2 version 3.3.2	R CRAN	https://cran.r-project.org/web/packages/ggplot2/index.html
R package pheatmap version 1.0.12	R CRAN	https://cran.r-project.org/web/packages/pheatmap/index.html
R package ggraph version 2.0.3	R CRAN	https://cran.r-project.org/web/packages/ggraph/index.html
R package igraph version 1.2.4.2	R CRAN	https://cran.r-project.org/web/packages/igraph/index.html
R package ggpubr version 0.2.5	R CRAN	https://cran.r-project.org/web/packages/ggpubr/index.html
R package ggrepel version 0.8.2	R CRAN	https://cran.r-project.org/web/packages/ggrepel/index.html
R package circlize version 0.4.8	R CRAN	https://cran.r-project.org/web/packages/circlize/index.html
R package reticulate version 1.16	R CRAN	https://cran.r-project.org/web/packages/reticulate/index.html
STAR-FINDer Single Cell and Spatial Transcriptomics Data Portal	This study	https://simmonslab.shinyapps.io/FetalAtlasDataPortal

### Resource availability

#### Lead contact

Further information and request for resources and reagents should be directed to and will be fulfilled by the Lead Contact, Alison Simmons (alison.simmons@imm.ox.ac.uk).

#### Materials availability

This study did not generate any unique reagents.

#### Data and code availability

The accession numbers for the raw and processed data used for this study was deposited on GEO and is publicly available (ST: GEO: GSE158328 and scRNA-seq GEO: GSE158702). Original data supplementary to the analysis highlighted in this manuscript have been deposited to Mendeley Data (DOI: 10.17632/gncg57p5x9.2) and are also publicly available (Fawkner-Corbett et al., 2020) https://doi.org/10.17632/gncg57p5x9.2

### Experimental models and subject details

#### Human samples

##### Tissue handling

Tissues from fetal intestine were initially collected and processed at the Human Developmental Biology Resource(HDBR), London. Following written informed consent and anonymisation (HBDR project 200462, REC: 18/LO/0822) samples were collected and intestinal tissue dissected or isolated. Intestinal tissue was placed in Lebowitz medium (L-15, Sigma) on ice, and transferred immediately to Oxford for further processing.

For adult intestinal samples, written consent was obtained (REC reference: 18/WM/0237) from patients undergoing non-emergency intestinal surgery with resection for IBD (e.g., stricture) or colorectal cancer. In both instances sampling was performed clear of inflammatory or oncological processes at the site of stoma formation. Samples were places in RPMI (Sigma) on ice and processed immediately in a similar manner to fetal tissue with one additional step to dissect the outer muscle under magnification.

#### Sample information

An overview of all samples used in the study for development of the atlas is deposited within supplementary materials and details age, genotype/gender alongside tissue processing and experimental multiplex barcoding where appropriate - (Fawkner-Corbett et al., 2020 Tab 1 “1.Sample Overview”). Although replication was not possible in individual samples due to their digestion, samples for scRNA-seq atlas were selected across a range of gestational ages to aim to provide n ≥ 2 biological replicates at each gestational age and a spread across developmental time (Figure 1B). No randomization or blinding of samples was performed. A power-calculation was not performed prior to study as samples were processed based on tissue quality, location and availability. No sequenced samples were excluded from the analysis. Exclusion criteria for individual cells and spots is described below, under “10X scRNA-Seq Data Analysis” and “Spatial Transcriptomics Data Analysis” sections.

### Method details

All fetal intestinal tissues were examined to identify anatomical landmarks (stomach, Meckel’s diverticulum, and/or appendix) and if present tissues from Terminal Ileum (TI), proximal colon and distal colon were separated for processing. In low gestation samples (≤12pcw) where only a small amount of colonic tissue remained, the entire tissue would be processed as “hindgut” without a proximal/distal division. TI was sampled by taking < 2cm upstream of appendix; similarly, in early gestation sampling was performed from the region upstream of the appendix or hindgut, due to small size of samples at these time points this tissue was also termed distal SI as it may extend past the TI. In samples that were piecemeal in nature and anatomical markings were unclear, a cryopreserved tissue area would have an adjacent paraffin fixed part of the lumen processed for H&E stain and discussed with a pediatric pathologist before consideration for scRNA-seq processing.

Tissue was washed in cold PBS and laid open and then either; proceeded directly to digestion; was cryo-preserved in CryostorCS10 (Sigma Aldrich) for later digestion as per manufacturer’s instructions; fixed in 10% neutral buffered formalin for 48 hours and then processed for paraffin embedding; or was embedded in OCT sectioning media (Thermo Scientific) by submersion in isopentane (2-methylbutane, Sigma Aldrich) pre-cooled to −80C in dry ice for ST.

#### Human intestinal scRNA-seq dissociation protocol

Digestion of intestinal tissue for scRNA-seq was performed by adapting previously reported protocols using and EDTA chelation of epithelial crypts and bulk digestion of remnant crypt depleted tissue; or in an initial experiment full digestion without crypt (Kinchen et al., 2018; Parikh et al., 2019).

Samples were processed fresh or, if cryopreserved thawed for 2 minutes in 37° waterbath then washed in 30 mL of DMEM(High glucose, Sigma Aldrich) with Penicilln and Streptomycin, HEPES (GIBCO) and 5% Fetal Calf Serum (Sigma Aldrich) before centrifugation and processing the tissue pellet in the same manner. All samples were cut into small (< 0.2cm) pieces. Following optimization, where a bulk digestion method of one step full-tissue digestion using umbilical cord digestion kit (Milteyni Biotech) of all tissue as per manufacturers instructions, the optimum method for digestion was identified for the rest of the atlas. This consisted of a crypt chelation protocol as previously described (Parikh et al., 2019) with a modification of increasing EDTA concentration in digestion media to 5mM and for two digestion steps of 20 minute in a waterbath at 37°C, with agitation and collection of the supernatant after each incubation. The isolated epithelial crypts in the supernatant were processed to a single cell suspension with TypLE Express (GIBCO) and DNase 50μg/ml (Sigma) for 45 minutes while the remaining crypt-depleted tissue was digested with Umbilical Cord Dissociation kit (Milteyni Biotec) at 37°C with regular agitation using a blunt needle and syringe. Once a single cell suspension was obtained for both the epithelial (EpCAM+) and non-epithelial (EpCAM-) fraction they were processed in tandem and washed with DMEM (high glucose, Sigma Aldrich) substituted with 5% fetal calf serum, filtered through a 70 μM and 40 μM filter, counted twice to ensure high viability (Countess II, Thermo Fisher) and up to 500,000 viable cells brought forward for hash-tag (HTO) staining. This method was validated for purity and viability using flow cytometry on n = 4 samples before proceeding to scRNA-seq (Viability [DAPI negative % of all cells] mean 90.7%, SD 2.2% EpCAM+ fraction / 96.3%, SD 1.8%; EpCAM- fraction; Epithelial [EpCAM/CD326 positive % of all live single cells] mean 93.1%, SD 5.0% EpCAM+ fraction / 5.7%, SD 4.7% EpCAM- fraction; Immune [CD45 positive % of all live single cells] mean 0.85%, SD 0.41% EpCAM+ fraction / 3.5%, SD 2.2% EpCAM- fraction; Stromal [CD90 positive % of all live single cells] mean 0.72%, SD 0.5% EpCAM+ fraction/ 81.9%, SD 7.3% EpCAM- fraction).

HTO antibody staining was performed on each single cell digested compartment. Antibody conjugated oligonucleotides were either commercially obtained (Total-seq, Biolegend) or conjugated in house to CD326 (EpCAM, 324229, Biolegend) which was divided in 10 μg aliquots to 9 unique oligonucleotides using iEDDA-click chemistry with tag sequences (HTO 1-9) the same as in published methods (Stoeckius et al., 2018). Reagents for the conjugation were obtained from; Sigma Aldrich (10x Borate buffered Saline, Dimethyl Sulfoxide and 1M Glycine); Bio-Rad (Micro bio-spin P-6 Gel columns; Biomers Ltd (Trans-cyclooctane PEG4 labeled oligos) and protocol undertaken as per online step by step protocol to enable scRNA-seq compatible antibodies (Stoeckius and Hao., 2020).

Isolated cells from each experimental condition were stained with an HTO antibody. The HTO stain was either with commercial antibody in bulk or EpCAM- cells from compartment method; commercial and in-house for EpCAM+ cells compartment method, as we found EpCAM+ in low gestation labeled poorly with HTO single tags (likely due to lower expression of target protein B2M (Figure S1B). Respective staining was performed for 30 minutes at 4°C and then washed with PBS supplemented with 0.04% BSA, counted, re-suspended at a concentration of 1,000 cells/μl. Stained samples were kept separately on ice, and pooled immediately before loading for scRNA-seq.

#### Droplet based scRNA-seq

Following digestion, samples underwent droplet based scRNA-seq using the 10x Chromium single cell platform (10x Genomics, 3′ v3 chemistry, Rev C) as detailed by manufacturer, with some modifications.

In an initial experiment cells were tagged with commercial HTO-antibodies (Biolegend) and a comparison of digestion was run from the same intestinal tissue. This highlighted that a proportion of epithelial cells were challenging to demultiplex due to low antibody staining (Figures S1B–S1H). Following this, experiments were undertaken with compartment method of dissociation, single HTO antibody in the non-epithelial compartment and dual HTO antibody staining in the epithelium.

Cells from individual experimental conditions were pooled and loaded as per manufacturers guidance (10x Genomics) with 30-37,000 cells and 3-9 HTO conditions per reaction (Fawkner-Corbett et al., 2020).Generation of gel beads in emulsion (GEMs), barcoding, GEM-reverse transcription, clean up, complimentary DNA amplification, library construction and index PCR followed manufacturers guidance (10x Genomics, 3′ v3 chemistry, Rev C). Modifications included the addition of a HTO additive primer at initial cDNA amplification, and the HTO-supernatant being removed during SPRI bead clean up with small-length fragments being taken and processed separately as reported previously (Stoeckius et al., 2018) to generate a small-length HTO library for sequencing using the online step-by-step protocol with 10 cycles of PCR library amplification (Stoeckius, 2015). Custom oligonucleotides for the modified steps were obtained from Sigma Aldrich with sequences identical to the step-by-step protocol (Fawkner-Corbett et al., 2020).

Sample quality of RNA and HTO library was assessed on Bioanalyzer Tapestation (Agilent) and concentration on Qubit 3.0 (Thermofisher). The final libraries were pooled and analyzed on either a Novaseq6000 or Nextseq (Illumina) using 150 base-pair paired-end reads with sequencing depth determined on cell recovery estimate or quantification of HTO run before gene expression (GEX) library with an aimed read depth of 40,000 (non-epithelial) or 50,000 reads/cell (epithelial) and HTO library sequencing depth at 5% of GEX library.

#### Spatial transcriptomics

For samples undergoing ST fetal and adult tissue was processed into sectioning blocks as described above. Blocks were stored in −80C in an air-tight container. Before undertaking a full protocol tissue freezing method was tested for RNA quality with RIN > 7.0 (RNA pico, Agilent) and a tissue optimization experiment (10x Genomics, Visium Spatial Tissue Optimization, Rev A) was performed with imaging of fluorescence footprint on InCell 6000 Analyzer (GE Healthcare) and image analysis performed in Fiji (ImageJ v2.0.0) identifying 18 minutes as optimum permeabilization time.

Fetal and adult samples were then processed for full ST experiment as per manufacturer’s instructions (10x Genomics, Visium Spatial, Rev B) being cut in a pre-cooled cryostat at 10 μM thickness onto four 6.5mm x 6.5mm capture areas with 5000 oligo-barcoded spots. For some samples (Adult, 12pcw TI and Colon) n = 2 technical replicates were performed. Slides then underwent fixation and H&E staining with immediate imaging on Aperio Scanscope (Leica Biosystems) at 40x magnification. Tissue underwent permeabilization with proprietary enzyme (18 minutes), reverse transcription and second strand synthesis performed on the slide with cDNA quantification undertaken with qRT-PCR using KAPA SYBR FAST-qPCR kit (KAPA Biosystems) and analyzed on the QuantStudio 7-Flex system (ThermoFisher). qRT-PCR results (Cq value at 25% of peak fluorescence) informed cDNA amplification.

Following library construction as per manufacturer’s instructions ST libraries were quantified using the KAPA-Illumina PCR quantification kit (KAPA Biosystems) and pooled at 4nM concentration with a sample ratio corresponding to the surface area of tissue coverage obtained from the H&E imaging. Pooled libraries were sequenced on a NextSeq (Illumina) using 150 base-pair paired-end dual-indexed set up (High output, v 2.5, Illumina) loaded at a concentration of 1.8pM. Four slides were sequenced to a manufacturer recommended depth of ∼50,000 reads per tissue covered spot (mean achieved: 52,714 reads). Four subsequent slides were deep sequenced to the depth of 362,034, 364,566, 143,014 and 183,966 mean reads per tissue-covered spot in order to increase sequencing saturation and detection rates of low expression transcripts.

This generated a dataset of 9330 tissue-covered spots with an average of 2480 genes detected per spot in each slide (3877 mean genes for deep sequencing slides). Each spot covered a 55μm area with a 100μm center-to-center distance, which should be expected to encompass 6-10 total cells.

Areas highlighted as anatomical reference points (e.g., myenteric plexus, Figure 2B) had images discussed with a pediatric pathologist (D.F.) before annotation.

#### Flow cytometry analysis

For analysis of cell dissociation efficiency, before undertaking scRNA-seq, cells in single-cell suspension as previously described were stained with EpCAM (PE-Cy7, Milteyni 1:50), CD90 (FITC, Biolegend 1:75), CD45 (APC, Milteyni 1:50) antibodies as appropriate for 30 minutes in PBS supplemented with 1% Bovine Serum Albumin. Stained cells were washed and anti-DNA staining (4’,6’-diamidino-2-phenylidole (DAPI), BD Biosciences, 1:1500) performed for 5 minutes before processing on Attune NxT flow cytometer (ThermoFisher) with compensation performed on single stained cells. Flow cytometry data was analyzed with FlowJo (FlowJo v10.5.3) and once purity and viability of dissociation protocols confirmed subsequent samples would proceed directly to single cell sequencing.

#### Immunohistochemistry, *in situ* hybridization and haemotoxylin and eosin staining

For immunohistochemistry (IHC), paraffin embedded tissue blocks were cut at a thickness of 5um onto slides which were deparaffinized by passing through an ethanol gradient and heat induced epitope retrieval (HIER) performed at 96°C for 25mins in ph6 (citrate) or ph9 (TRIS-EDTA) buffer. Peroxidase was blocked and a species-specific primary antibody was added for 90 minutes at room temperature (Key Resources Table) before addition of species specific secondary. Stain was developed with either single or dual peroxidase / alkaline phosphatase kits (Impact, Vector) as per manufacturer’s instructions.

*In situ* hybridization (ISH) was performed using kits and probes from Advanced Cell Diagnostics (ACD) which used oligonucleotide probes (Key Resources Table) to RNA targets and development with RNAScope 2.5HD assay brown (cat 310035) as per manufacturer’s instructions with incubations steps of processed paraffin embedded tissue performed in HybEz oven (ACD, cat321461).

H&E staining was carried out by deparaffinisation in a similar manner and then addition of stains as described by H&E kit (Vector laboratories). Haemotoxylin was added for 1 minute before mounting IHC stains when single stains were used.

### Quantification and statistical analysis

#### Detailed characterization of intestinal scRNA-seq clusters

Single cell RNA-sequencing results were analyzed as an entire complement which readily identified cellular compartments (Figure 1C). The atlas was divided into compartments based on known morphological features which could be recapitulated with key marker genes for separate analyses. Compartments included differential expression of epithelial (*EPCAM, FABP1),* fibroblast (*THY1, COL1A2, VIM)*, myofibroblast (*VIM, FOXF1, TAGLN*), endothelium (*PECAM1, CDH5, CLDN5*), pericyte (*KCNJ8, ABCC9, TGS5*), neural/glial (*PHOX2B, HAND2, TUBB2B)*, immune (*PTPRC*) and muscle (*MYH11, ACTG2*) genes (Figure 1D). In totality 101 clusters were annotated representing different cell types or cell states throughout the intestine (Figure 1G). A full overview of compartmental and intra-compartmental annotation is outlined in Table S1 and complete genes list deposited at Fawkner-Corbett et al., 2020.

#### Epithelial characterization

15 epithelial clusters were identified and annotated by their gene expression profile as; Enteroendocrine cells (*CHGA, TPH1* and *NEUROD1*) (Gehart et al., 2019), transit-amplifying cells (*MKI67, UBE2C* and *TOP2A*) (Parikh et al., 2019), enterocytes (*FABP2, CEACAM1* and *EPCAM*) (Dalerba et al., 2011), goblet cells (*MUC2, SPDEF* and *WFDC2*) (McCauley and Guasch, 2015; Parikh et al., 2019) and stem cells (*LGR5, ASCL2* and *SMOC2*) (Barker, 2014). One cluster expressed enterocyte (*CA7, CA4*) genes along with a distinct transcriptional signature including *BEST4* and *OTOP2* which was in keeping with recently described BEST4/OTOP2 cells (Parikh et al., 2019). Alongside the secretory population, a cluster expressed genes described in EEC (*NEUROD1*) (Li et al., 2019) and goblet cell (*DLL1*) progenitors (van Es et al., 2012). *FOXA2* expression was high which matched a combined secretory lineage and so was annotated as secretory progenitor (Ye and Kaestner, 2009). Remaining differences in clusters were annotated as per locational (proximal, distal) differences in samples, or maturity of enterocytes with a spectrum of maturity markers (*AQP8, FABP2*) from TA cells to higher expression in mature cells.

In this initial characterization intra-cluster expression of specific genes, most notably EEC hormones, could still be seen. Thus the secretory lineage was also analyzed in isolation this generated 11 clusters which included an EEC progenitor marked by *NEUROG3* and other genes similar to transcriptional analysis of organoid modeling (Beumer et al., 2020; Gehart et al., 2019). Goblet cells divided into 3 clusters which appeared to represent a spectrum of goblet cell-types from cycling (Goblet 1, *UBE2C, MKI67*) to primitive stem-cell like (Goblet 2, *MYC, TRAB2A* and low expression *LGR5*) and relatively higher expression of mature goblet cell markers (Goblet 3, *MUC2, TFF3, SPDEF*). The EECs divided into 6 cluster with key hormones mimicking previously reported signatures (Beumer et al., 2020; Gehart et al., 2019); Somatostatin (*SST,* D-cells), serotonin (TPH1, Enterochromaffin cells), cholecystokinin (*CCK*, I cells), Peptide YY/ glucagon (*PYY*, *GCG* L-Cells), Neurotensin (*NTS*, N cells) and co-expression of Motilin and Ghrelin was seen in the same cluster and annotated as A-Cell/M-Cells similar to other studies. The final cluster was small intestinal specific and expressed defensins (*DEFA5, DEFA6*) in keeping with Paneth cells (Haber et al., 2017).

#### Fibroblast characterization

Fibroblast cells separated computationally into 16 distinct clusters. Two clusters demonstrated high expression of cell cycle phase genes and so were annotated Fibroblast G2M phase (*MKI67, TOP2A*) and Fibroblast S-phase (*PCLAF, TYMS*) in accordance with these. There was a small distinct number of contaminating erythroid cells (*HBB, GYPA*) that were clearly discrete and excluded from downstream analysis. The remaining fibroblast clusters were divided into fibroblast subtypes which mimicked those seen in scRNA-seq of adult intestinal tissue and were annotated as such (Kinchen et al., 2018); fibroblast S1 (*ADAMDEC1, FABP5*), S2 (*FRZB, BMP5*), S3 (*C7, ASPN*) and S4 (*CCL19, C3*). Within these further transcriptional diversity was seen and annotations were then based on locational differences, such as in S2 (Distal S2, Proximal S2 1 and Proximal S2 2), or on key genes that differentiated sub-types – *COL6A5+* and *IFIT3* population in S1, and *CXCL13* and *CCL21* population in S4 respectively. A relatively larger proportion of fibroblasts were of the S3 type and this was reflected in 5 clusters expressing S3 genes but differing in gene expression, two populations were annotated on higher expression of transcription factors *HAND1* and EBF (*EBF3, EBF3*) respectively. One S3 population had relatively lower expression of S3 marker genes and was placed between the fibroblast progenitor and mature S3 populations on trajectory analysis thus annotating these as “S3 Transitional” (Figure S6B).

There was one cluster that did not express fibroblast subset-specific markers and had relatively lower expression of mature fibroblast genes (*THY1, IGFBP4*). Alongside this, the frequency of cells in this cluster was higher in early (< 12pcw) time points and one of the highly expressed genes *HMGA2* has been shown to be involved in embryonic fibroblast formation (Tessari et al., 2003) leading to the annotation of “Fibroblast progenitor.” Similar to this, an adjacent cluster had a relatively lower expression of S3 type genes (*OGN, FBLN1*) and was represented in earlier time-points leading to the annotation of S3 progenitor.

#### Myofibroblast/ mesothelial characterization

Within the myofibroblast cells there was a population representing cells within the G2M (*CENPF, MKI67*) and S-phase (*PCLAF, TYMS*) of cell cycle and were thus annotated accordingly. In the remaining clusters, 2 were characterized by *WT1* and *MSLN* which are both reported as markers of mesothelial layer in the early formation of abdominal viscera (Rinkevich et al., 2012; Wilm et al., 2005), these clusters were annotated based on their marker genes; *SOX6* and *IL18* mesothelium which may arise due to locational contribution. The remaining two clusters expressed more classical myofibroblast genes such as *NKX-3* and *ACTG2* (McLin et al., 2009; Roulis and Flavell, 2016)but one had higher expression of mature markers (*MYH11, ACTA2*) and contained cells from later in developmental time, leading to the annotations of “myofibroblast progenitor” and “myofibroblast.”

#### Endothelial characterization

The 11 clusters that comprised the EC compartment could be divided into five venous clusters which expressed known EC vein specific genes such as *ACKR1* and *VWF* (Thiriot et al., 2017)and four arterial clusters expressing genes such as *GJA4* and *HEY1* (Kalucka et al., 2020).

Further annotation of these clusters related to expression of genes reported to reflect the size of the vessel in mice, which appeared to be recapitulated in human development. In venous EC’s genes reported to be more highly expressed in capillaries (*CD83, RGCC*) supported the annotation of “venous capillaries (CP)” and contrasted to large vein specific genes such as *ACKR1* or *ADGRG6* (Kalucka et al., 2020; Thiriot et al., 2017).

Similarly, in arterial clusters a spectrum of gene expression was seen ranging in expression of large arterial vessel genes (*HEY1, GJA5)* through to genes reported in capillary endothelial cells (*VWA1*, *RBP7*) (Buschmann et al., 2010; Hu et al., 2017; Kalucka et al., 2020). Annotation in turn reflected this with large(L), medium sized (M) or two capillary (CP1, CP2) endothelial cell clusters.

Alongside cells expressing cell cycle genes (*MKI67, CENPF*) one further group of cells was distinct from the vascular ECs and had specific expression of genes such as *PROX1, PDPN* and very high expression of *LYVE1* which are known to be markers of lymphatic ECs in the murine intestine and so annotation reflected this (Kim et al., 2007).

#### Pericyte characterization

Within the pericyte compartment eight clusters of cells were identified, with two expressing genes identifying them as cycling (*MKI67, PCLAF*). One distinct group of cells was present mainly within the colon and had high expression of genes that have been reported to be seen in pericytes involved in angiogenesis such as PRRX1 and *PROCR* so was termed angiogenic colonic pericytes (Higuchi et al., 2015; Yu et al., 2016). The remaining pericytes formed a continuity of cells which appeared to reflect a maturation gradient from an undifferentiated pericyte population with relatively low expression of pericyte marker genes through to cells expressing muscle related genes (*MYH11, ACTG2*, “contractile pericytes”), or fibroblast-like genes (*THY1, SPON2, BMPER* “BMPER+ pericytes”). Alongside the undifferentiated pericyte cluster, a second group of cells expressed *THBS1* that is seen in early differentiating pericytes (Niimi et al., 2019) and this in addition to the majority of cells being from early (< 12pcw) time points led to the annotation of “pericyte progenitors.” *WNT6* was specifically expressed in cells intermediately placed between the undifferentiated and contractile pericytes, this gene has been shown in animal models to be important in muscle differentiation (Lavery et al., 2008) and this may be reflected in intestinal pericyte development in this intermediate stage between differentiating pericytes (with loss of *THBS1*) and so this cluster was annotated “WNT6 pericytes.”

#### Neural characterization

Along with a cycling cluster (*MKI67, CENPF*) there were 5 clusters of cells that expressed genes matching glial (*SOX10, S100B*), and 7 neural (*ELAVL4, CHRNA3)* cell types respectively.

Within glial cells there was a cluster with relatively lower expression of mature glial markers, represented by predominantly early time point cells and also expressed *PHOX2B* which is seen in enteric nervous system (ENS) progenitors and was thus termed “glial progenitors” (Lasrado et al., 2017). *ENTPD2* expression has previously been reported in intra-ganglionic glia and so cells expressing this were annotated as such (Gulbransen and Sharkey, 2012). A separate cluster had relatively high expression of *TGFB1*, which is expressed by subsets of glial that are closely adherent to the epithelial surface. The function of this cluster was further supported by gene ontology (GO) enrichment (Fawkner-Corbett et al., 2020) which highlighted terms including “regulation of epithelial cell proliferation” and “epithelial cell proliferation.” This cluster was thus termed “submucosal glia” and a separate cluster with lower expression of similar genes, an increase in early-time point cells and glial-specific expression of *HAND2* (reported to be important in glial precursors in mural intestinal ENS development) led to these cells being annotated as “submucosal precursors” (Lei and Howard, 2011). The final glial cell cluster was transcriptionally distinct from other glial cell types and exhibited high expression of genes involved in immune infiltrate formation (*FGL2*), T cell transduction (*MAL*) and expressed TGF-beta receptors (*TGFBR3*) highlighting the possibility of these cells interacting with immune cells (Dong et al., 2018). Furthermore this cluster expressed *GFRA3, ARTN* and retinoic acid receptor (*RXRG*) which matched the description of a glial subset in mice that is vital for Peyers patch development and led to this cluster being termed “lymphoid associated glial” (Veiga-Fernandes et al., 2007).

For neuronal clusters; interneurons were identified by expression of tachkinin (*TAC1*) and enkephalin (*PENK*), inhibitory motor neuron by expression of nitric oxide (*NOS1*) and *VIP* and excitory motor neurons by expression of *CASZ1* (Gulbransen and Sharkey, 2012; Monteiro et al., 2016), *SLC5A7* and *GFRA2* – the latter two genes were identified as part of the core transcriptional signature of excitory neurons in a recent scRNA-seq study of the ENS (Drokhlyansky et al., 2020). A cluster adjacent to the inhibitory motor cells expressed similar genes such as *SCGN* and *NOS1,* but at a lower level and was thus annotated as “inhibitory motor neuron precursor.” Two cluster showed mature neuronal markers and specifically expressed a number of markers that matched enteroendocrine circuits including neuropeptides (*SST*, *NPY*) and their receptors (*SSTR2, NPY2R*) so were termed neuroendocrine 1 and neuroendocrine 2. The final neural cluster had a relatively lower expression of neuronal markers such as *ELAV4* and expressed genes seen in primitive neuronal progenitors such as *DLL3* and *DLL4* so was annotated as “ENS progenitors” (Nelson et al., 2013).

#### Immune characterization

Pooling of all cells expressing pan-immune markers (*PTPRC*) identified 12 clusters. Alongside a cycling cluster (*MKI67, CENPF*) cells were further subdivided based on 5 lymphoid clusters (Adaptive and innate) 5 of myeloid origin (monocyte, macrophage, dendritic cell(DCs)) with a final cluster exhibiting a distinct signature in keeping with intestinal mast cells (*TPSAB1, TPSB2, CPA3*) (Parikh et al., 2019; Smillie et al., 2019).

Specifically, monocytes were identified by high expression of *CD14* in comparison to macrophages as well as *FCN1* a key gene expressed in colonic monocytes in colorectal cancer (Zhang et al., 2020) alongside transcription of Myeloid Inhibitory C-type Lectin like receptor (MICL encoded by *CLEC12A*) reported in this cell type (Marshall et al., 2004). Macrophages exhibited expression or reported genes and markers such as *MERTK* (Bain and Schridde, 2018)as well as Cathepsin C and D (*CTSC, CTSD*) (Martin et al., 2019). Interestingly there were two macrophage clusters which differed in expression of *SPP1,* a recently described gene identifying polarize macrophages termed SPP1+ Macrophages (Zhang et al., 2017), and the second expressed genes involved in complement formation (*C1QA, C1QC*) and *IL1B* at a higher level – in keeping with a classically activated (M1) phenotype (Azizi et al., 2018; Zhang et al., 2020).

Two clusters represented DCs, by expression of the pan-marker *ETV6* (Robbins et al., 2008) were divided into conventional DC (*FLT3*) and plasmocytic DCs (*PTCRA, LILRA4*) (Martin et al., 2019; Villani et al., 2017).

Within the lymphoid compartment, B cells were seen with expression of genes including *CD19*, and clustered into cells which had more mature B cells markers (*CD19hi, CD79A, IGHD*) and a cluster with a more naive appearance; relatively lower expression of *CD19,* expressing light chain *IGLL1*, and other genes reported in naive B cells such as *VPREB3* (Bendall et al., 2014; Hay et al., 2018). This and their appearance in early gestational age led to the annotation ‘Pre-B cell’ (O’Byrne et al., 2019).

Three cell clusters had signatures in keeping with innate lymphoid cells, the first expressing markers of a naive T cell phenotype (*CD3G, CCR7, CD27*) and another representing a Gamma delta cells (*TRDC*) and NK cells (*IL2RB*) (Martin et al., 2019). The final cluster expressed *KIT* highlighting it as a ILC cell which together with *KLRB1*, *RORC* and *ID2* demonstrated this to be of the ILC-3 subtype (Martin et al., 2019). The expression signature, timing of appearance and interaction with other compartments highlighted this cell type as representing the Lymphoid Tissue Inducer (LTi) population of ILC-3 cells.

#### Muscle characterization

Intestinal muscle cell compartment comprised of 11 clusters. Two were readily identifiable as cycling in G2M (*MKI67, CENPF*) or S-phase (*PCLAF, TYMS*) based on transcriptional signature. Within the compartment there was a clear temporal divide, with two clusters being represented by predominantly early time point (< 12pcw) samples. The first expressed *PDGFRA* and *KIT* which was in keeping with PDGFRA+ interstitial cells (Sanders et al., 2016), it was also closely related to a second cluster expressing *KIT* alongside *ANO1* and *SPON2* – resembling the Interstitial cells of Cajal (Lee et al., 2017). The second early time-point cluster expressed primitive genes such as *HOX5C* alongside myocardin (*MYOCD*) which is a critical factor in early smooth muscle differentiation (Du et al., 2003). Along with low expression of *FOXF2*, a marker identifying the intestines internal musculature (Bolte et al., 2015), highlighted this cluster as an internal muscle progenitor. Similar to IM progenitor *FOXF2* was expressed in a gradient across a number of clusters with highest expression in two clusters termed proximal and distal muscularis mucosae, supported by expression of *IGF1*, *HHIP* and *NOG* which are known to be key to smooth muscle and lamina propria development mechanisms respectively (Kuemmerle, 2006; Mifflin et al., 2011). Another inner muscle cluster specifically expressed *PMAIP1 (*aka NOXA) which is important in smooth muscle differentiation, so annotation reflected this (Bai et al., 2008). The other two IM clusters had intermediate expression of *FOXF2* and a lower expression of myofibroblast-like marker *VIM,* when compared to muscularis mucosae clusters, suggesting a slightly more peripheral placement and so was termed IM proximal and IM distal based on locational differences. The final two clusters were negative for the internal muscular markers *FOXF2* and *ACTC1* and expressed *IGFBP5* which found at a higher level in outer intestinal muscle thus leading to the annotations proximal and distal outer muscularis (OM) based on locational differences (Gurdziel et al., 2016; Zimmermann et al., 2001).

#### Raw sequencing data processing

All raw sequencing data was converted to from bcl to fastq format using Illumina bcl2fastq software, version 2.20.0.422, with tolerance of up to one mismatch in sample index barcode. Raw sequence reads were quality checked using FastQC software (Andrews, 2010).

#### Raw 10X scRNA-seq and spatial transcriptomics raw sequence data processing

Human hg38 reference genome analysis set was downloaded from the University of California Santa Cruz (UCSC) ftp site (Kuhn et al., 2013). Human hg38 reference genome Ensembl gene annotations were obtained using the UCSC Table Browser Tool (Karolchik et al., 2004).

For each sequenced scRNA-Seq pool, Cellranger software (version 3.1.0) from 10 × Genomics (https://support.10xgenomics.com/single-cell-gene-expression/software/downloads/latest) was used to process, align and summarize unique molecular identifier (UMI) counts against hg38 human reference genome.

Similarly, Spaceranger (version 1.0.0) software from 10X Genomics was used to process, align and summarize UMI counts against hg38 human reference genome for each spot on the Visium spatial transcriptomics array.

Corresponding antibody libraries were processed separately using CITE-Seq Count (version 1.4.3) to obtain antibody tag UMI counts for each cell barcode using the following parameters: -cbf 1 -cbl 16 -umif 17 -umil 28 -T 8 -cells 200000. Read UMI counts were summarized using 16-base barcodes for TotalSeq antibody libraries and 12-base barcodes for the in-house conjugated antibody libraries (see Fawkner-Corbett et al., 2020] for barcode sequences). Antibody UMI count matrices were then further filtered against the 10X cellular barcode whitelist for the corresponding 10x version 3.0 chemistry.

#### 10X scRNA-seq data analysis

Raw UMI count matrices were imported into R for further processing. For each scRNA-Seq sample, cell calling was performed using ‘emptyDrops’ (Lun et al., 2019) function from DropletUtils (version 1.4.3) on the full raw count matrices in order to distinguish cells from empty droplets containing only ambient RNA. Raw count matrices were corrected for Illumina index swapping using ‘swappedDrops’ (Griffiths et al., 2018). This identified 140,264 non-empty droplets across all single cell pools.

Furthermore, droplet barcodes for which a high percentage of total UMIs originated from mitochondrial RNAs were filtered out, as well as low total UMI count barcodes. These thresholds were derived individually for cells within each compartment following an initial clustering solution of all cells by examining and thresholding empirical distributions within each compartment, as total RNA content (notably higher in endothelial and myeloid cell populations) and mitochondrial RNA content (notably higher in epithelial cells) are highly cell type dependent.

For each individual pool, Seurat (Butler et al., 2018) R package (version 3.1.5.9900) was used to normalize expression values for total UMI counts per cell. Highly variable genes were identified by fitting the mean-variance relationship and dimensionality reduction was performed using principal-component analysis. Scree plots were used to determine principal components to use for clustering analyses for each pool. Cells were then clustered using Louvain algorithm for modularity optimization using kNN graph as input. Cell clusters were visualized using UMAP algorithm (McInnes et al., 2018) with principal components as input and n.neighbors = 30, spread = 1 and min.dist = 0.1.

Cells from separate pools were merged and pool batch effect signal was corrected using harmony (version 1.0) algorithm (Korsunsky et al., 2019). Merged cell clustering and visualization of cells from all pools was performed as before using Louvain and UMAP algorithms, using harmony dimensionality reduction as input instead of principal components. Merged pool clusters were compared with cell types obtained from individual pools to ensure cell type heterogeneity was not lost due to batch correction.

Merged cell data was then divided into compartments based on clustering analysis and marker gene expression, as outlined above. Cells from epithelial, endothelial, pericyte, muscularis, neural, fibroblast, immune, myofibroblast and mesothelial compartments were subset for further analysis. For each compartment, we carried out compartment-specific QC, batch correction and clustering analyses as described above.

Cell identities for compartment sub-clusters were assigned based on markers outlined above.

#### Hashed sample de-multiplexing

Non-epithelial cell pools were demultiplexed as follows. HTO antibody UMI count matrices were initially filtered to keep only 10x cellular barcodes from droplets passing QC based on mRNA expression profiles, as described above. For each hashed pool, the filtered matrix was then used to demultiplex samples as originally described in Stoeckius et al. (2018) and implemented in HTODemux function in r package Seurat. Briefly, counts were normalized using centered log ratio transformation and for each pool, an initial clustering solution was obtained using clara k-mediods clustering with k = 1 + number of samples in the pool. For each cluster/hash ID, we then fit a negative binomial distribution and define a positive threshold at 99th percentile of the recovered normalized UMI counts for the hashtag, with cells below this threshold considered negative for the tag. Cell identity is then assigned based on individual hashtag thresholds and multiplets defined as cells positive for multiple tags. Multiplets were filtered out. Cells negative for all hash tags formed a minor fraction and were also filtered out, following inspection of their mRNA-cluster distributions. Untagged cells correlated with lower total mRNA content cells and did not segregate with any particular cluster and thus likely contained unstained/dying cells or free nuclei that have lost their cytoplasm during sample processing. Demultiplexed cells were visualized as tSNE plots from Euclidian distance matrixes.

The above procedure was modified to incorporate dual-labeling of cells in epithelial cell pools as follows. TotalSeq and in-house hashing antibody UMI counts matrices were normalized using centered log ratio transformation and the initial clustering solution was obtained using the matrices of both tags. For each cluster, we then fit a negative binomial distribution separately for TotalSeq and in-house tags and derived positive and negative thresholds based on 99^th^ percentile as before. For each cell, we compared identities assigned using in-house tags and TotalSeq antibody tags, observing disagreement between cell sample-of-origin identities in 6.3%- 10.5% of cells. The majority of cases where the two tags disagreed were accounted by either a) cells from lower gestation samples (Figures S1E and S1F) which could be identified by the in-house tag but showed poor staining by the TotalSeq antibody; or b) non-epithelial contaminant cells which were negative for the in-house tag (Figure S1H, see mRNA expression of epithelial *EPCAM* and stromal *THY1*/CD90 superimposed over tag-derived clusters).

In order to assign identities to these cells, we separated the antibody matrices into cells where in-house and TotalSeq tags agree. For each pool, we then trained a kNN classifier using the cells where in-house and TotalSeq tags agree, and following 10-fold cross-validation of the training data we then assigned sample-of-origin identities to the remaining cells based on their nearest neighbors.

In each case, we then further examined whether sample demultiplexing was correct by ascertaining that the expression of sex-specific genes, such as XIST, segregated correctly with sample-of-origin assignments.

Using the above procedure, we classified all non-empty droplets (140,264) as singlets (98,866), untagged/negative (18,670) or multiplets (22,728), thus placing the overall seen multiplet rate at 16.2%. The 98,866 demultiplexed singlets were retained and subjected to further QC per compartment as described in 10X scRNA-Seq Data Analysis to retain 76,592 high quality, confidently demultiplexed singlets.

#### Spatial transcriptomics data analysis

Raw UMI count spot matrices, images, spot-image coordinates and scale factors were imported into R. Spot matrix was filtered out to keep only spots overlaying tissue sections. Raw UMI counts were normalized using regularised negative binomial regression (SC Transform) (Hafemeister and Satija, 2019) to better account for variability in total spot RNA content. Dimensionality reduction was performed using PCA and for each slide, scree plots were examined the determine the optimum number of principal components to use in downstream clustering analyses.

Clustering was performed using Louvain clustering algorithm as before (resolution = 0.5) and clusters were visualized using UMAP algorithm as before. Clusters distributions were visualized in spatial context over H&E images with spot size scaling factor of 1.6 used throughout.

Cell type prediction probabilities were calculated for each spot using factor analysis via FindTransferAnchors and TransferData functions in Seurat. In addition to single cell data generated here, we also used single cell populations obtained from adult tissue reference datasets (Kinchen et al., 2018; Parikh et al., 2019; Smillie et al., 2019; Wang et al., 2020) (GEO: GSE114374, GEO: GSE116222, DUOS-000110 & GEO:GSE125970) to predict spot content for all slides.

For all predictions, pairwise cell type prediction probability score Pearson correlations were calculated using all tissue covered spots in a given slide in order broadly to assess spatial cell type co-occurrence within the same spot. Correlation r matrices were clustered using hierarchical clustering and significant correlations (adjusted p value < 0.05) were visualized in cell type pairwise heatmaps.

#### Spatial transcriptomics spot distance-based analyses

For distance-based analyses, intestinal tissue in H&E images was first demarcated up as follows. In adult tissue sample slides, muscularis mucosa was marked and used as a reference point throughout. For fetal tissue sample slides, serosal membrane was marked up of intact cross-sections in each slide. In each slide, we then filtered out spots covering sections of tissue containing artifacts, including tissue folds. Spots covering the inner-most parts of the lumen were also filtered out, as these were found to contain mRNA signatures consistent with apoptotic epithelial cells, including very high mitochondrial content and low spot library complexity. For each remaining spot, we then calculated Euclidean distance from the center pixel of the spot to the nearest marked up pixel. Adult tissue sample slides were segmented prior to this to account for helical positioning of the tissue. Spots in the adult mucosa were assigned positive distance values to indicate distance toward the lumen, and spots in the submucosa were assigned negative distance values to indicate distance away from the muscularis mucosa. Spots in fetal tissue slides were all assigned positive distance values to indicate distance toward the lumen. As the distance between spots between slides is uniform, this then provides a relative distance measure that is comparable between tissue sections.

Distance measures were then used to examine cell type score distributions relative to tissue depth in the intestine using density over distance plots.

In order to identify significant depth-varying genes in tissue sections, we fit generalized linear negative binomial models to each gene, testing whether gene expression is dependant on spot distance. In order to test for non-linear dependencies, we constructed natural splines (with 3 degrees of freedom) for spot distance. P values were corrected for multiple testing using Benjamini-Hochberg correction and genes with < 5% FDR were considered significantly co-varying with distance in each slide.

Distance-varying genes were subsequently clustered using hierarchical clustering based on their expression patterns to group similarly expressed genes together. Each tree was cut using ‘cutree’ function in R into k = 2 or k = 3 clusters and Gene Ontology enrichment analysis was performed for each gene cluster, as described below.

Smoothed gene expression patterns over distance were visualized using heatmaps obtained from loess regression fits of expression across distance.

#### Spatial co-localization of receptor-ligand pairs

To identify spatially co-localizing receptor-ligand pairs, we tested all putative receptor-ligand pairs identified from our single cell analysis. First, for each receptor-ligand pair, as before we fit a generalized linear model to test whether the expression of the receptor is dependant on the expression of the ligand in individual spots in each given slide. After multiple-testing correction (Benjami-Hochberg), pairs with adjusted p value of < 0.05 and a positive coefficient were considered as spatially co-localizing.

As some receptor-ligand pairs may signal over larger distances, we next sought the repeat the analysis by also including adjacent spots in the analysis. For each spot, we first calculated a distance-smoothed expression matrix as the mean expression in any given spot and spots immediately surrounding it. We then repeated the co-localization analysis as described above.

#### Marker gene detection and differential expression analyses

For all marker gene expression, we used R packages Seurat and MAST for statistical testing (Finak et al., 2015). Briefly, for each identified cluster, we compared the cells within that cluster versus all other cells. To identify markers within sub-populations in compartments, we compared cells from each cluster versus all other cells within a given compartment. Location-specific genes were identified within each cluster by comparing cells from TI or colon tissue samples. In cases where we observed location-driven clustering, we performed comparisons with the nearest location-matched counterpart cluster (e.g., distal stem cells versus proximal stem cells; distal S2 versus proximal S2). In each case, confounding sources of variation stemming from cellular detection rate, pool batch effects and donor genotype were included in the model formula as covariates.

For visualizing and thresholding cell type specificity, we calculated gene AUC scores for all cell types using area under the curve analysis for each gene as implemented in Seurat FindMarkers function (test = ROC).

In order to identify time-course varying differentially expressed genes in scRNA-Seq data, we used zero inflated negative binomial models. For each comparison (cluster-level or compartment level), we used the ‘zinbwave’ function from R package “zinbwave” (Risso et al., 2018) to fit single cell count data for each gene and compute their observational weights. DESeq2 (Love et al., 2014)was then used to perform differential expression analyses, with zinbwave observational-level weights used in parameter estimation step, with the following non-default parameters: sfType = ”poscounts” and minmu = 1e-6. To identify genes that may co-vary with developmental time course in a non-linear fashion, as before we used natural cubic splines function (degrees of freedom = 3) of post-conceptual weeks time course in the model fit together with batch co-variates. Then, a likelihood ratio test was used to compare to the reduced model which excluded the time course variables. P values were corrected using Benjamini-Hochberg multiple testing correction and genes were considered significant at < 5% FDR. FDR values lower than 2.2e-16 were summarized as < 2.2e-16 in the text, while full precision values are provided in supplemental data.

#### Identification of time-point and location associated single cell clusters

To identify cell clusters significantly associated with location or time point differences, for each cluster for each sample we first normalized the total number of cells detected within the compartment in that sample. To test for compartment-level differences, we normalized to the total number of cells detected for each sample in each 10x pool. Then, the percentages of cells in each sample were compared using a two-sided Wilcoxon test. P values < 0.05 were considered as significantly different. For location-specific clusters, we compared location-matched samples only.

#### Transcription factor module analysis

R package “SCENIC” workflow (Aibar et al., 2017) was used to detect active transcription factor modules in CD8+ cells. Single cell gene expression matrix was first filtered to exclude all genes detected in fewer than 10 total cells. The RcisTarget database containing transcription factor motif scores for gene promoters and around transcription start sites for hg38 human reference genome were downloaded from (https://resources.aertslab.org/cistarget/databases/homo_sapiens/hg38/refseq_r80/mc9nr/gene_based/) and the expression matrix was further filtered to only include genes available in the RcisTarget database. The remaining genes were used to compute a gene-gene correlation matrix for co-expression module detection using random forest based GENIE3 algorithm and R package ‘SCENIC’ was used to perform transcription factor network analysis to detect co-expression modules enriched for target genes of each candidate TF from RcisTarget database. AUCell package was used to compute a score for each TF module in each individual cell. First, for each cell we use expression matrix to compute gene expression rankings in each cell using “AUCell_buildRankings” function with default parameters. Downstream transcription factor target gene sets were then used to score each cell, where for each gene set and each cell, area under the curve (AUC) value was computed (“AUCell_calcAUC” function) based on gene expression rankings, where AUC value represents the fraction of genes within the top ranking genes for each cell that are defined as part of the TF gene set.

In order to identify cell type and condition-specific transcription factor modules, we fit generalized linear models to test condition or cell-type dependence of transcription factor AUC values. As AUC values were also significantly dependant on cell gene detection rate, we further included the gene detection rate as a blocking co-variate in our models. Obtained p values were subject to Benjamini-Hochberg multiple testing correction.

To create a transcription factor “decision tree” and identify transcription factors which define closely related cell sub-populations, we first calculated the mean AUC value for each transcription factor module in each cluster and performed hierarchical clustering analysis. We then iteratively traversed the branches of the cluster tree at each split, evaluating the transcription factor specificity differences between all the child cell type clusters in both branches. The lowest level nodes were evaluated first, such that a transcription factor module would not be considered specific to a higher-level branch split if it was already assigned as a differentiator of a lower-level split. For each split, we visualized the top two transcription factor modules as hierarchical clustering dendrogram labels.

#### Trajectory and RNA velocity analysis

RNA velocity estimates can be used to predict the directionality of a biological process such as cell differentiation in a continuous cell gradient by comparing the dynamics of spliced and unspliced/nascent mRNAs. We first calculated raw UMI counts matrices for spliced and unspliced mRNAs from BAM files in each 10x pool. Matrices were imported into R and merged with compartment level UMAP embeddings. R packages velocyto.R (La Manno et al., 2018) and SeuratWrappers were then used to estimate RNA velocity vectors using batch-corrected harmony dimensionality reduction, with velocity parameters kCells = 25, fit.quantile = 0.2 and deltaT = 1 and visualization parameters n = 200, grid.n = 40, arrow.scale = 3, min.grid.cell.mass = 0.5 and scale = ”sqrt.”

Trajectories on compartment embeddings were calculated using Monocle 3 algorithm (Qiu et al., 2017). Dimension-reduced data from integrated and batch corrected pools (as previously described) for each compartment were transferred into Monocle 3 cell_data_set objects. Cells were reclustered to enable learning of disjoint graphs in multiple partitions and the trajectory graph was learnt, allowing for closed loops and multiple partitions. For each compartment, the start of the trajectory was selected as the node nearest to the identified progenitor populations and trajectory pseudotime was then computed from the selected starting node.

In order to better understand the higher level connectivity structure of 101 single cell populations identified in our atlas, we employed a partition-based graph abstraction algorithm (Wolf et al., 2019) (scanpy accessed via reticulate (version 1.16) and SeuratWrappers (version 0.1.0) in R) using batch-corrected, dimension-reduced data as defined above and cell identities as defined above from the clustering analyses. The resultant transition confidence matrix was further filtered out to keep only edges above 0.4 confidence threshold. R package ‘igraph’ (version 1.2.4.2) was used to generate the graph abstraction layouts, using edge confidence-weighted force-directed layout and R package ggraph (version 2.0.3) was used to visualize the results.

#### Receptor-ligand analysis

To perform receptor-ligand analysis, we downloaded the receptor-ligand database compiled by Cabello-Aguilar et al. (2020). We then scored each receptor-ligand pair in our dataset in a pairwise manner for cells in each of our identified cell populations using the scoring procedure as described in Cabello-Aguilar et al. (2020). Both paracrine and autocrine interactions were computed across all pairwise cluster combinations (101x101 = 10201 possible cluster pairs), resulting in signaling network spanning 4452511 total putative interactions across all clusters involving 2552 unique receptor ligand pairings were identified (654 unique ligands and 646 unique receptors). We further prioritised interactions by assigning a cell type specificity AUC score to each receptor and ligand for every interaction. To visualize all putative receptor-ligand interactions between any given pair of clusters, we used circos plots via R package ‘circlize’ (version 0.4.8). Receptor-ligand interactions with an interaction score lower than 0.5 and cell type specificity AUC values lower than 0.7 were filtered out for clarity. Similarly, individual receptor-ligand pair cell type networks were constructed from interaction edges meeting the same criteria. R package ‘ggraph’ (version 2.0.3) was used to visualize these networks.

#### Cycling cell type classification analysis

Cycling cells in G2M and S phases in each compartment segregated into clusters and were identified by G2M and S phase marker genes (e.g., MKI67). In order to identify which cell sub-population phenotypes are the most representative of each cell in G2M or S phase clusters, for each compartment we divided the dataset into cycling and G1 phase cell populations and used the G1-stage cells to predict the closest cell label of the cells in G2M/S-phase using the cell label transfer method described in Stuart et al. (2019) by computing a cell type probability matrix for all cycling cells using non-cycling cells as reference. Each cycling cell was then assigned the label of nearest non-cycling cell in each compartment based on the maximum probability score.

As described in the “*Identification of Time-point and Location Associated Single Cell Clusters”* section above, we repeated the analysis to identify cell populations which are the most proliferative at various times during developmental time course by comparing the proportions of predicted cell types in each sample in cycling cell populations over time course. By comparing the relative proportions of cell types in cycling and non-cycling cell clusters, we could identify the cell types which showed relative enrichment in cycling populations and thus identify which cell populations are more likely to serve as proliferative progenitors to more differentiated cell sub-types.

#### Morphogen module identification

We curated a list of morphogens expressed in the fetal intestine from pathways known to that contribute to patterning, organogenesis and crypt formation encompassing their ligands, receptors, co-receptors, antagonists and transcription factors. This included Hedgehog, NOTCH, WNT, HIPPO, RTK, TGF-Beta, FEGF and EGFR signaling pathways (Fawkner-Corbett et al., 2020). Using the curated gene list, we performed weighted gene co-expression network analysis using R package WGCNA (Langfelder and Horvath, 2008). Briefly, a signed network was constructed by first estimating the soft power parameters (8 for single cell analysis and 12 for spatial transcriptomics analysis) and used to compute a pairwise distance matrix for the morphogens. Dynamic hydrid tree cut method (parameters: minModuleSize = 6, deepSplit = 1) was used to partition the genes into modules.

We then computed a score for each detected module for all spots in spatial transcriptomics slides by averaging expression levels of each module in each spot and subtracting similarly aggregated expression of randomly selected control features, as implemented in R package Seurat.

In order to visualize morphogen pathway interactions and their modules, we downloaded high confidence interactions from STRING database (Szklarczyk et al., 2019) (version 11.0) for the morphogens. A network was constructed using R package igraph (version 1.2.4.2) and layed out using a force-directed layout. R package ‘ggraph’ (version 2.0.3) was used for all network visualizations.

#### Disease gene analysis

Human Phenotype Ontology (HPO) (Köhler et al., 2019) was curated by a clinical expert to select human disease phenotype terms pertaining to congenital or developmental gastrointestinal disease. We then used the curated HPO phenotypes and ontology annotations to link selected phenotypes to Online Mendelian Inheritance in Man (OMIM) and OrphaNet human diseases and their associated causative disease genes. We identified 749 disease genes linked to 18 phenotypes, 718 of which were found to be at least minimally expressed in our single cell data.

To identify highly cell type specific genes, we performed area under the curve analysis and calculated AUC values for each disease gene for each cluster. Disease genes with AUC values > 0.8 were considered highly cell type specific. Hierarchical clustering analysis was performed to highlight cell type specific disease gene hubs, which were visualized as mean cell type expression heatmaps using R package “pheatmap” (version 1.0.12).

To identify developmental time-course varying disease genes, we used differential expression analyses as described above.

#### Gene Ontology enrichment analysis

Gene Ontology and pathway enrichment analyses were performed using the ‘clusterProfiler’ R package (Yu et al., 2012). Annotation Dbi R package “org.Hs.eg.db” was used to map gene identifiers. In each case, all expressed/detected genes were used as a background control and gene sets were tested for over-representation in cluster markers or differentially expressed genes by computing enrichment p values (‘enricher’ R function, default parameters) from the hypergeometric distribution of total genes in the background gene set, the number of genes within background that are annotated with the gene set, the size of the gene set and the number of genes within cluster marker/differentially expressed genes list annotated with the gene set. Hypergeometric p value was adjusted in each case for multiple testing using Benjamini-Hochberg correction as before. The results were visualized as dot plots and emap plots using ‘clusterProfiler’, ‘enrichPlot’ and ‘ggplot2′ R packages.

### Additional resources

#### Single cell RNA-seq and spatial transcriptomics data portal

Interactive analyzed data with searchable functions has been provided as an online resource – the Spatio-Temporal Analysis Resource of Fetal Intestinal Development: STAR-FINDer (https://simmonslab.shinyapps.io/FetalAtlasDataPortal/). STAR-FINDer has features including: gene expression, ST, gene regulator networks, trajectory analysis, time-course differences; morphogen expression; RL interactions.

The data portal template was developed using R Shiny framework. The data portal follows a modular design, with a standalone module and UI for each individual data modules: gene expression queries, spatial transcriptomics queries, gene regulatory networks, trajectory analyses, cell type proportion differences, morphogen co-expression modules and receptor-ligand interactions.

All large data has been pre-computed and stored in a back-end SQLite database independently for each module. Small scale data is read into memory at the start of the application to facilitate quicker queries.

All plotting is performed using R packages ggplot2 (version 3.3.2), pheatmap (version 1.0.12), ggraph (version 2.0.3), ggrepel (version 0.8.2), ggpubr (version 0.2.5) and circlize (version 0.4.8).

## Supplemental information

Table S1. Overview of key genes from scRNA-seq data, divided by each cell compartment, which exhibit relatively high or specific expression in each cell types used for characterization annotation, related to Figure 1
