# Supplementary material for: Spatiotemporal analysis of human intestinal development at single-cell resolution
Source: Cell. 2021 Feb 4;184(3):810–826.e23. doi: 10.1016/j.cell.2020.12.016 (PMC7864098; doi:10.1016/j.cell.2020.12.016)
Supplement: Table S1. Overview of key genes from scRNA-seq data, divided by each cell compartment, which exhibit relatively high or specific expression in each cell types used for characterization annotation, related to Figure 1 [file mmc1.pdf]

Cell, Volume *184*

Supplemental Information

Spatiotemporal analysis of human intestinal development at single-cell resolution

David Fawkner-Corbett, Agne Antanaviciute, Kaushal Parikh, Marta Jagielowicz, Ana Sousa Gerós, Tarun Gupta, Neil Ashley, Doran Khamis, Darren Fowler, Edward Morrissey, Chris Cunningham, Paul R.V. Johnson, Hashem Koohy, and Alison Simmons

Table S1: Overview of key genes from scRNA-seq data, divided by each cell compartment, that exhibit relatively high or specific expression in each cell types used for characterisation annotation, related to Figure 1 ([a]further sub-division of secretory cells is also shown, [b]Comparison made as proportion of each compartment cells, if cluster is locational specific comparison of time-point difference made only in that location and overall. Locational and time-point differences: Wilcox rank test, p-value <0.05 *; p-value <0.01 **; p-value <0.01 ***, p-value <0.001****)

Compartment	Compartment Genes	Cell Type	Key Genes (Cell Type)	Cluster Name (Graph abstraction number)	Key Genes (Cluster)	Locational Differences	Time-point Differences[b]
**Epithelial**	*EPCAM, FABP1*	Stem	*LGR5, ASCL2, MYC*	Proximal Stem Cells (1)	*CASP1, GRIA4, PLA2G2A*	Increased in small intestine (****)	No significant differences
				Distal Stem Cells (2)	*LEFTY1, PTGDR, SMOC2*	Increased in Colon (****)	Increases over time in colon and overall
		TA	*MKI67, TOP2A, UBE2C*	Proximal TA (3)	*CENPM, TK1, UBE2T*	Increased in small intestine (****)	Decreases over time overall
				Distal TA (4)	*HMGB3, MND1, TACC3*	Increased in colon (****)	No significant differences
		Secretory[a]	*FOXA2, INSM1, HES6*	Secretory Progenitors (5-15)	*ISL1, NEUROG3, GHRL*	Increased in small intestine (**)	No significant differences
				EECs (5-15)	*CHGA, TPH1, NEUROD1*	No locational preference	Increases over time in colon, TI and overall
				Goblet cells (5-15)	*MUC2, SPINK4, WFDC2*	No locational preference	Increases over time in colon and overall
		Absorptive	*FABP2, APOE* (proximal), *FAM3D* (distal)	Proximal Progenitors (16)	*FGG, SOX11, FGB*	Increased in small intestine (**)	Decreases over time in TI and overall
				Proximal Early Enterocytes (17)	*TF, AFP, VTN*	Increased in small intestine (**)	No significant differences
				Proximal Enterocytes (18)	*CCL25, HEBP1, OSR2*	Increased in small intestine (***)	No significant differences
				Proximal Mature Enterocytes (19)	*APOC3, ACE, FABP2*	Increased in small intestine (**)	Increases over time in TI and overall
				Distal Absorptive (20)	*CA12, HMGCS2, SLC13A2*	Increased in colon (****)	No significant differences
				Distal Enterocytes (21)	*SLC26A2, FABP1, CKB*	Increased in colon (****)	No significant differences
				Distal Mature Enterocytes (22)	*AQP8, FABP6, IL32*	Increased in colon (****)	Increases over time in colon and overall
			N/A	BEST4/OTOP2 cells (23)	*BEST4, OTOP2, CA7*	No locational preference	Increases over time in TI
**Secretory[a]**	*N/A*	Progenitor	N/A	*NEUROG3*+ Progenitor (5)	*NEUROG3, CLPS, MDK*	N/A	
		Goblet	*MUC2, TFF3*	Goblet 1(6)	*UBE2C, PBK, MKI67*		
				Goblet 2 (7)	*TRABD2A, PTGDR, MYC*		
				Goblet 3 (8)	*SPDEF, WFDC2, ATOH1*		
		EEC	Cluster specific	A-Cells/M-Cells (9)	*CLEC4G, MLN, GHRL*		
				D-Cells (10)	*SST, HHEX, PCP4*		
				Enterochromaffin Cells (11)	*TPH1, RXFP4, CHGA*		
				I-Cells (12)	*CCK, GC, TDO2*		
				L-Cells (13)	*PYY, GCG, UCN3*		
				N-Cells (14)	*NTS, HOPX, MEP1A*		
		Paneth	N/A	Paneth (15)	*PRSS2, DEFA5, DEFA6*		
**Fibroblast**	*THY1, COL1A2, VIM*	Cycling	N/A	Fibroblast G2M phase (24)	*CENPF, TOP2A, MKI67*	No locational preference	Decreases over time in colon, TI and overall
			N/A	Fibroblast S-Phase (25)	*PCLAF, TYMS, GINS2*	No locational preference	Decreases over time in colon, TI and overall
		Progenitor	N/A	Fibroblast progenitor (26)	*HMGA2, MBP, THY1(low)*	No locational preference	Decreases over time in colon, TI and overall
		S1	*ADAMDEC1, FABP5, CTSC*	S1 (27)	*PDLIM3, COL28A1, TFPI2*	Increased in colon (**)	Increases over time in colon and overall
				S1 *COL6A5*+ (28)	*COL6A5, LXN, WNT4*	Increased in small intestine (***)	Increases over time in TI
				S1 *IFIT3*+ (29)	*IFIT3, CLEC2B, GPX3*	No locational preference	Increases over time in colon, TI and overall
		S2	*FRZB, SOX6, F3*	Proximal S2 1 (30)	*NPY, CXCL10, BMP2*	Increased in small intestine (****)	Increases over time in TI
				Proximal S2 2 (31)	*MMP11, INSC, BMP3*	Increased in small intestine (****)	No significant differences

				Distal S2 (32)	*POSTN, ALKAL2, WNT5B*	Increased in colon (****)	Increases over time in colon and overall
		S3	*C7, ASPN, OGN*	S3 progenitor (33)	*OSR2, NR2F1, PTN(low)*	No locational preference	Increases then decreases over time in colon and overall
				S3 Transitional (34)	*GREM1, GDF10, FBLN1*	No locational preference	Increases over time in colon, TI and overall
				S3 (35)	*RAMP1, SCN7A, SCUBE2*	No locational preference	Increases over time in colon, TI and overall
				S3 EBF+ (36)	*PRRX1, EBF3, EBF2*	Increased in colon (*)	Increases over time in colon and overall
				S3 HAND1+ (37)	*COL9A3, DLK1, HAND1*	Increased in colon(*)	Increases over time in colon and overall
		S4	*CCL19, C3, CD74*	S4 CXL13+ (38)	*CXCL13, TNFSF11, CD24*	Increased in small intestine (*)	Increases over time in TI and overall
				S4 CCL21+ (39)	*CCL21, OLFM3, PTGDS*	No locational preference	Increases over time in colon, TI and overall
**Myofibroblast /Mesothelium**	*VIM, FOXF1, TAGLN, RSPO2, RGS5(-)*	Cycling	N/A	Myofibroblast G2M phase (40)	*MKI67, CENPF, TOP2A*	No locational preference	No significant differences
			N/A	Myofibroblast S-phase (41)	*PCLAF, TYMS, GINS2*	No locational preference	Increases over time overall
		Myofibroblast	*THY1, NKX2-3, ACTG2*	Myofibroblast progenitors(42)	*ADAMDEC1, MFAP4, ADAM28*	No locational preference	Decreases over time in colon and overall
				Myofibroblasts (43)	*ACTA2, MYH11, GREM2*	No locational preference	Increases over time in colon, TI and overall
		Mesothelium	*MSLN, WT1, RSPO1*	Mesothelium *IL18*+(44)	*IL18, GATA6, WNT10A*	No locational preference	No significant differences
				Mesothelium *SOX6*+ (45)	*SOX6, HPGD, STX2*	Increased in colon (**)	No significant differences
**Endothelium**	*PECAM1, CDH5, CLDN5*	Cycling	N/A	Endothelium G2M-phase (46)	*MKI67, CENPF, TOP2A*	No locational preference	Decreases over time in colon, TI and overall
				Endothelium S-phase (47)	*PCLAF, TYMS, GINS2*	No locational preference	Decreases over time in colon and overall
		Arterial	*GJA4, IGFBP3,, UNC5B*	Arterial (L) (48)	*HEY1, GJA5, ITGB4*	No locational preference	Increases over time in colon and overall
				Arterial (M) (49)	*UNC5B, DLL4, PRND*	No locational preference	Increases over time in colon
				Arterial (CP) (50)	*VWA1, AFG1L, EDNRB*	No locational preference	Increases over time in colon, TI and overall
				Arterial (CP) 2 (51)	*CHST1, LGALS1, IGFBP3*	No locational preference	Decreases over time in colon and overall
		Venous	*MADCAM1, APLNR, PRCP*	Venous (L) (52)	*ACKR1, VWF, ADGRG6*	No locational preference	Increases over time in colon and overall
				Venous (M) 1 (53)	*LRG1, PLVAP, STMN1(-)*	No locational preference	Increases over time in colon, TI and overall
				Venous (M) 2 (54)	*CALCRL, APLNR, PRCP*	No locational preference	Decreases over time overall
				Venous (CP) 1 (55)	*CCL2, CD69, RGCC*	No locational preference	Decreases over time in colon and overall
				Venous (CP) 2 (56)	*LDHB, JUNB(-), ZFP36(-)*	No locational preference	Decreases over time in colon, TI and overall
		Lymphatic	N/A	Lymphatic (57)	*LYVE1, PROX1, RELN*	No locational preference	Increases over time in colon and overall
**Pericytes**	*KCNJ8, ABCC9, RGS5, CSP4G*	Cycling	N/A	Pericyte G2M-phase (58)	*MKI67, CENPF, TOP2A*	No locational preference	Decreases over time in TI and overall
			N/A	Pericyte S-phase (59)	*PCLAF, TYMS, GINS2*	No locational preference	Decreases over time in colon and overall
		Pericytes	See compartment genes	Pericyte progenitors (60)	*THBS1, MBP,CEBPD*	No locational preference	Decreases over time in colon and overall
				Undifferentiated pericytes (61)	*CYGB, NFKBIA, TESC*	No locational preference	Increases over time in colon and overall
				WNT6+ pericytes (62)	*WNT6, GPAT2, THBS1(-)*	No locational preference	Increases over time in colon and overall
				Angiogenic pericyte (63)	*PROCR, EBF2, THBS4*	Increased in colon (**)	Increases over time in colon and overall
				BMPER+ pericytes (64)	*BMPER, CYGB, SPON2*	No locational preference	Increases over time in colon, TI and overall
				Contractile pericyte (65)	*ADIRF, MYH11, ACTA2*	No locational preference	Increases over time in colon and overall
**Neural**	*PHOX2B, HAND2, TUBB2B*	Cycling	N/A	Neural G2M & S-Phase (66)	*MKI67, CENPF, TOP2A*	No locational preference	Decreases in time in colon and overall
		Glial	*S100B, SOX10, PLP1*	Lymphoid associated glial (67)	*FGL2, GFRA3, RXRG*	Increased in colon (**)	Increases over time in colon and overall
				Intraganglionic glial (68)	*ENTPD2, SPRY1, SOX6*	No locational preference	Increases over time in colon, TI and overall
				Submucosal glia (69)	*CEBPD, SOCS3, ZFP36*	No locational preference	Increases in time in colon and overall
				Differentiating submucosal glial (70)	*BCAN, APOE, LAMP5*	No locational preference	Increases over time in colon and overall
				Glial progenitors (71)	*PHOX2B, HMGA2, CTGF*	No locational preference	Decreases over time in colon, TI and overall
		Neuronal	*ELAVL4, CHRNA3, GAP43*	ENS progenitors(72)	*DLL3, DLL1, ELAVL4(low)*	No locational preference	Decreases over time in colon and overall
				Neuroendocrine 1 (73)	*NEUROD6, SCGN, SSTR2, NPY2R*	No locational preference	Increases in time in colon and overall
				Neuroendocrine 2 (74)	*DGKG, SST, KCTD16*	No locational preference	Increases in time in colon, TI and overall

				Excitory motor neuron (75)	*CASZ1, RAMP1, GFRA2*	No locational preference	No significant differences
				Inhibitory motor neuron precursor (76)	*SCGN, CRABP1, NOS1, VIP, ADCYAP1*	No locational preference	Decreases over time in TI and overall
				Inhibitory motor neuron (77)	*NOS1, VIP, HTR2B, ETV1*	No locational preference	Increases then decreases in time in colon and overall
				Interneuron (78)	*PENK, ACHE, TAC1, ONECUT2*	No locational preference	Decreases over time
**Immune**	*PTPRC*	Macrophage	*MERTK, CTSC, CTSD, CD14 (hi)*	Macrophage (79)	*FGL2, HES1, SLC40A1*	Increased in colon (*)	Increases over time in colon and overall
				Macrophage SPP1+ (80)	*SPP1, RNASE1, FOLR2*	Increased in colon (*)	No significant differences
		Monocyte	*CD14 (lo)*	Monocyte (81)	*FCN1, S100A8, CLEC12A*	No locational preference	No significant differences
		Dendritic cell	*ETV6, FLT3*	pDCs (82)	*IL3RA, LILRA4, PTCRA*	No locational preference	No significant differences
				DCs (83)	*CD207, BATF3, CD1D*	No locational preference	No significant differences
		Adaptive lymphoid cells	*CD19, EBF1, PAX5*	Pre B Cell (84)	*IGLL1, VPREB1, RAG1*	No locational preference	No significant differences
				B cell (85)	*BANK1, CD79B, IGHD*	No locational preference	Increases over time in colon, TI and overall
		Innate lymphoid cells	*ETS1, TOX*	Naïve T Cell (86)	*CD3D, CD3G, CD27*	No locational preference	Increases over time in colon, TI and overall
				NKs and γδ (87)	*CD8A, IL2RB, TRDC*	Increased in small intestine (*)	No significant differences
				Type 3 ILCs (88)	*IL7R, ID2, RORC, KRT86*	No locational preference	Increases over time in colon and overall
		Eosinophils	N/A	Mast (89)	*TPSAB1, CPA3, MS4A2*	No locational preference	Increases over time in TI and overall. Decreases in colon.
		Cycling	N/A	Immune Cell cycle (90)	*MKI67, CENPF, TOP2A*	No locational preference	Decreases over time in colon, TI and overall
**Muscle**	*MYH11, ACTG2, TAGLN*	Cycling	N/A	Muscularis G2M phase (91)	*MKI67, CENPF, TOP2A*	No locational preference	Decreases then increases over time in colon
				Muscularis S-phase (92)	*PCLAF, TYMS, GINS2*	No locational preference	No significant differences
		Interstitial Cells	*KIT*	Interstitial Cells of Cajal / ICCs (93)	*ANO1, ETV1, SPON2*	No locational preference	No significant differences
				PDGFRA+ cells (94)	*PDGFRA, ITGA8, BMP4*	No locational preference	Decreases over time in colon and overall
		Outer muscularis	*FOXF2(-)*	OM (95)	*BCHE, CAPN3, IGFBP5*	No locational preference	Increases over time in colon and overall
		Inner muscularis (IM)	*FOXF2, ACTC1*	Proximal IM (96)	*IGFBP2, HOXC8, IGFBP7*	Increased in small intestine (****)	No significant differences
				Distal IM (97)	*PNCK, MORN5, AHNAK2*	Increased in colon (***)	Increases over time in colon and overall
				Proximal MM (98)	*PLCG2, NPNT, IGF1*	Increased in small intestine (****)	No significant differences
				Distal MM (99)	*PLN, MOXD1, TMEM158*	Increased in colon (***)	Increases over time in colon and overall
				IM *PMAIP1+* (100)	*PMAIP1, HMGCS1, KRT17*	No locational preference	Increases then decreases over time in colon and overall. Increases in TI.
		IM progenitor	*N/A*	IM progenitor (101)	*HOXC5, PLPP2, MYOCD*	No locational preference	Decreases over time overall.
